# Therapeutic applications of nanobiotechnology

**DOI:** 10.1186/s12951-023-01909-z

**Published:** 2023-05-06

**Authors:** Yogesh Dutt, Ramendra Pati Pandey, Mamta Dutt, Archana Gupta, Arpana Vibhuti, Jasmina Vidic, V. Samuel Raj, Chung-Ming Chang, Anjali Priyadarshini

**Affiliations:** 1 Department of Microbiology, SRM University, 39, Rajiv Gandhi Education City, Post Office P.S. Rai, Sonepat, Haryana 131029 India; 2 Mamta Dental Clinic, Opposite Sector 29, Main Badkhal Road, Faridabad, Haryana 121002 India; 3 Department of Biotechnology, SRM University, 39, Rajiv Gandhi Education City, Post Office P.S. Rai, Sonepat, Haryana 131029 India; 4 Université Paris-Saclay, Micalis Institute, INRAE, AgroParisTech, 78350 Jouy-en-Josas, France; 5 Master & Ph.D Program in Biotechnology Industry, Chang Gung University, No.259, Wenhua 1st Rd., Guishan Dist., Taoyuan City, 33302 Taiwan (ROC)

**Keywords:** Nanobiotechnology, Nanoparticles, Anticancer agents, Wound healing, Tissue regeneration

## Abstract

Nanobiotechnology, as a novel and more specialized branch of science, has provided a number of nanostructures such as nanoparticles, by utilizing the methods, techniques, and protocols of other branches of science. Due to the unique features and physiobiological characteristics, these nanostructures or nanocarriers have provided vast methods and therapeutic techniques, against microbial infections and cancers and for tissue regeneration, tissue engineering, and immunotherapies, and for gene therapies, through drug delivery systems. However, reduced carrying capacity, abrupt and non-targeted delivery, and solubility of therapeutic agents, can affect the therapeutic applications of these biotechnological products. In this article, we explored and discussed the prominent nanobiotechnological methods and products such as nanocarriers, highlighted the features and challenges associated with these products, and attempted to conclude if available nanostructures offer any scope of improvement or enhancement. We aimed to identify and emphasize the nanobiotechnological methods and products, with greater prospect and capacity for therapeutic improvements and enhancements. We found that novel nanocarriers and nanostructures, such as nanocomposites, micelles, hydrogels, microneedles, and artificial cells, can address the associated challenges and inherited drawbacks, with help of conjugations, sustained and stimuli-responsive release, ligand binding, and targeted delivery. We recommend that nanobiotechnology, despite having few challenges and drawbacks, offers immense opportunities that can be harnessed in delivering quality therapeutics with precision and prediction. We also recommend that, by exploring the branched domains more rigorously, bottlenecks and obstacles can also be addressed and resolved in return.

**Graphical Abstract:**

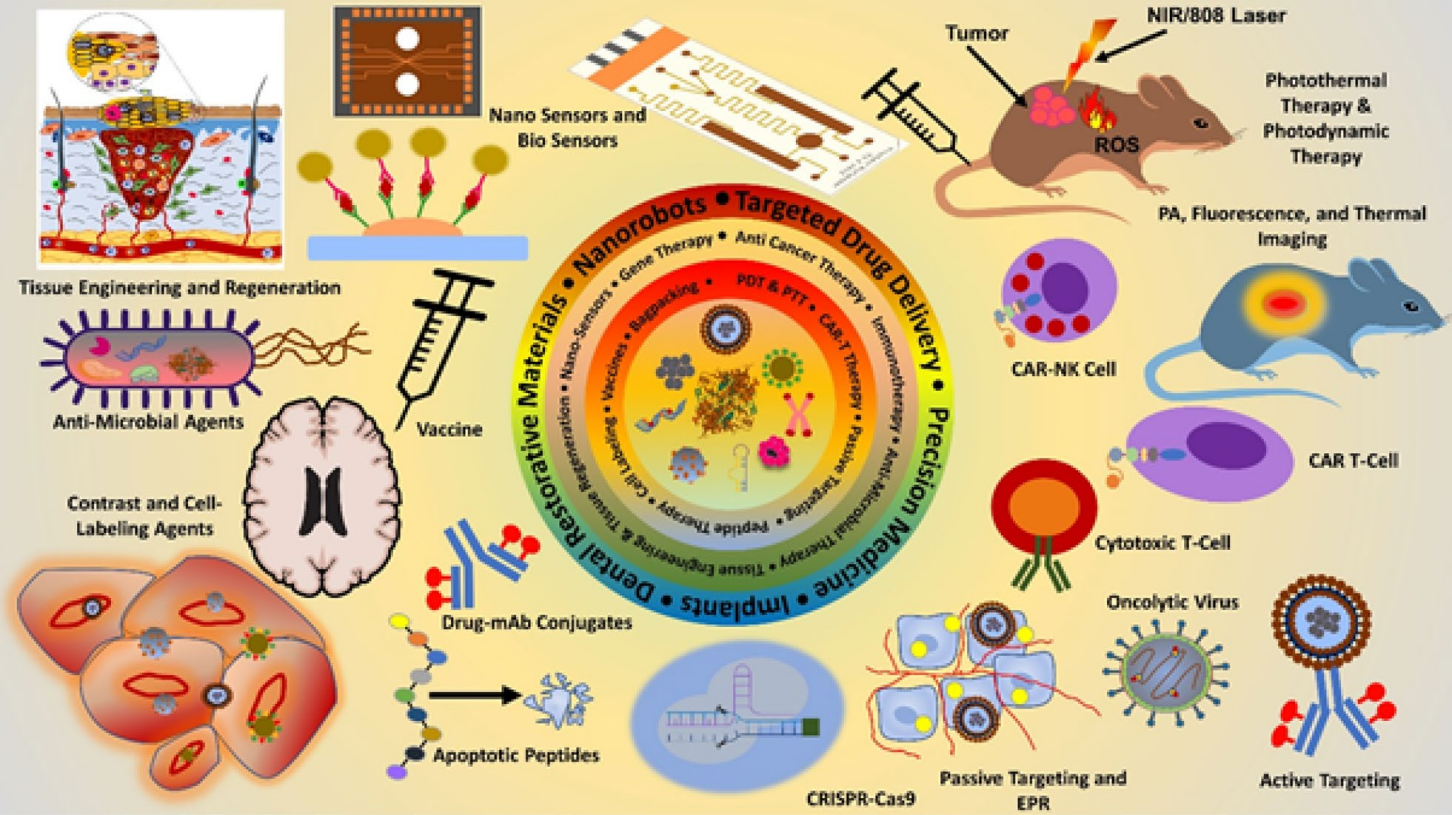

## Introduction

### Background

Concepts of nanotechnology or nanobiotechnology are not old, but originated from Richard Feynman's vision of building objects from bottom up at CIT in 1959 [1]; however, his concept was not taken seriously for next two decades until 1986 with publication of “Engines of Creation” by Eric Drexler mentioning approaches and vision of controlled manufacturing of products at molecular scale [2]. Nanotechnology is the design and fabrication of structures at atomic, molecular, or macromolecular levels by manipulating or modifying basic structure of materials efficiently in order to augment, modulate, or change the properties; due to the functions of cellular components at nanoscale level [3]. Nanotechnology was very suitable to be applied in biological sciences and conceptualised the term of nanobiotechnology. Nanobiotechnology came into existence as a novel and more specialized field or branch of science by amalgamation of methods, techniques, and protocols from other branches of science like nanotechnology, biology, and biochemistry; and amalgamation of these branches have resulted in formulation of unique and new methodologies as well as materials [4]. As a multi-strategic technique, nanobiotechnology was developed by amalgamation of nanotechnology and biotechnology in order to modify or improve the dynamics or properties of nanomaterials or nanoparticles (NPs); one of the prominent examples is target delivery of biomolecules or drugs through functionalized-nanoparticles (FNPs) to target tissue or organ [3].

### Rationale of nanobiotechnology

Due to extremely small size, NPs and other nanostructures can enter cells, interact with organelles, and yield distinct effects [4–6]; and due to that, nanostructures contribute significantly in drug-delivery system, contrast agents, photothermal effects, and imaging [3, 4, 7]. Prototypical features of nanostructures including NPs have given advantageous edge over conventional methods for theranostics of carcinomas and tumors; such leverages are due to the ability of NPs to reach target cells or tissue without diffusing to the adjacent areas [4]. Such typical features are not enjoyed with conventional diffusing or anti-cancer therapeutic agents and usually precipitate unwanted after-effects and cytotoxicity to normal healthy cells. Conventional contrast or therapeutic agents can target both cancerous as well as healthy cells, however targeted-NPs are formulated only to reach cells-of-interest [8]. Methods of nanobiotechnology might help in understanding the cellular pathways, signalling, and disease progression through identification of novel biomarkers and mechanisms of drug action efficiently; additionally, modification of nanomaterials has enabled scientists to conjugate bioactive molecules such as enzymes, photosensitizers, therapeutic drugs, and even nucleic acids with modified biomaterials [4]. Such advancements have opened new untapped potentials of nanobiotechnology in the areas of cancer diagnosis and prevention, antimicrobial therapies, and prevention of morbidities.

### Nanobiotechnology in general

**Fig. 1:**
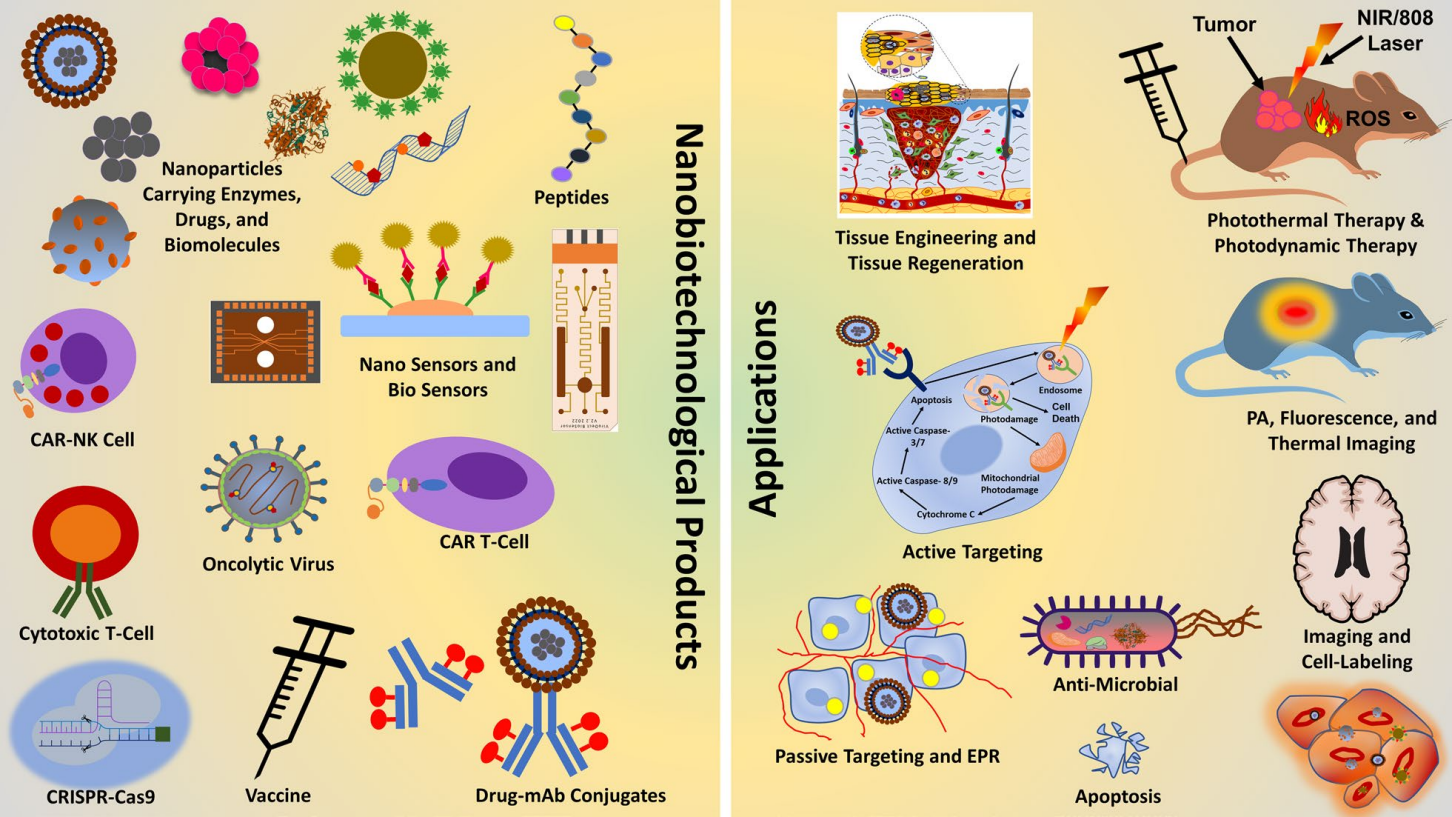
Nanobiotechnology and its applications. (Parts of the figure reproduced with permission from all the authors) [5, 6]

Nanobiotechnology is the design, fabrication, modulation, and uses of nanomaterials including nanoparticles (< 100 nm) and appliances made from these nanomaterials mainly nanocarriers or other drug delivery systems; this enables many conventional therapeutic agents to be used through repurposing [3]. As a prominent product of nanobiotechnology, NPs can protect therapeutic agents from enzymatic degradation and reticuloendothelial system (RES); also enhance the circulation time, thereby improve the chances for reaching target sites [4]. Nanobiotechnology (illustrated in Fig. 1) is the smart assimilation of techniques and methods from nanotechnology, biology, pharmacology, and physics for the development of novel nanomaterials and devices for therapeutic purpose with improved efficiency and applications; few of these nanomaterials applied in drug delivery systems, imaging, antimicrobial and anticancer therapies, in-vitro diagnostics with progressive improvements are nanoparticles, nanotubes, and nanofibers among others [3].

### Nanoparticles

**Fig. 2:**
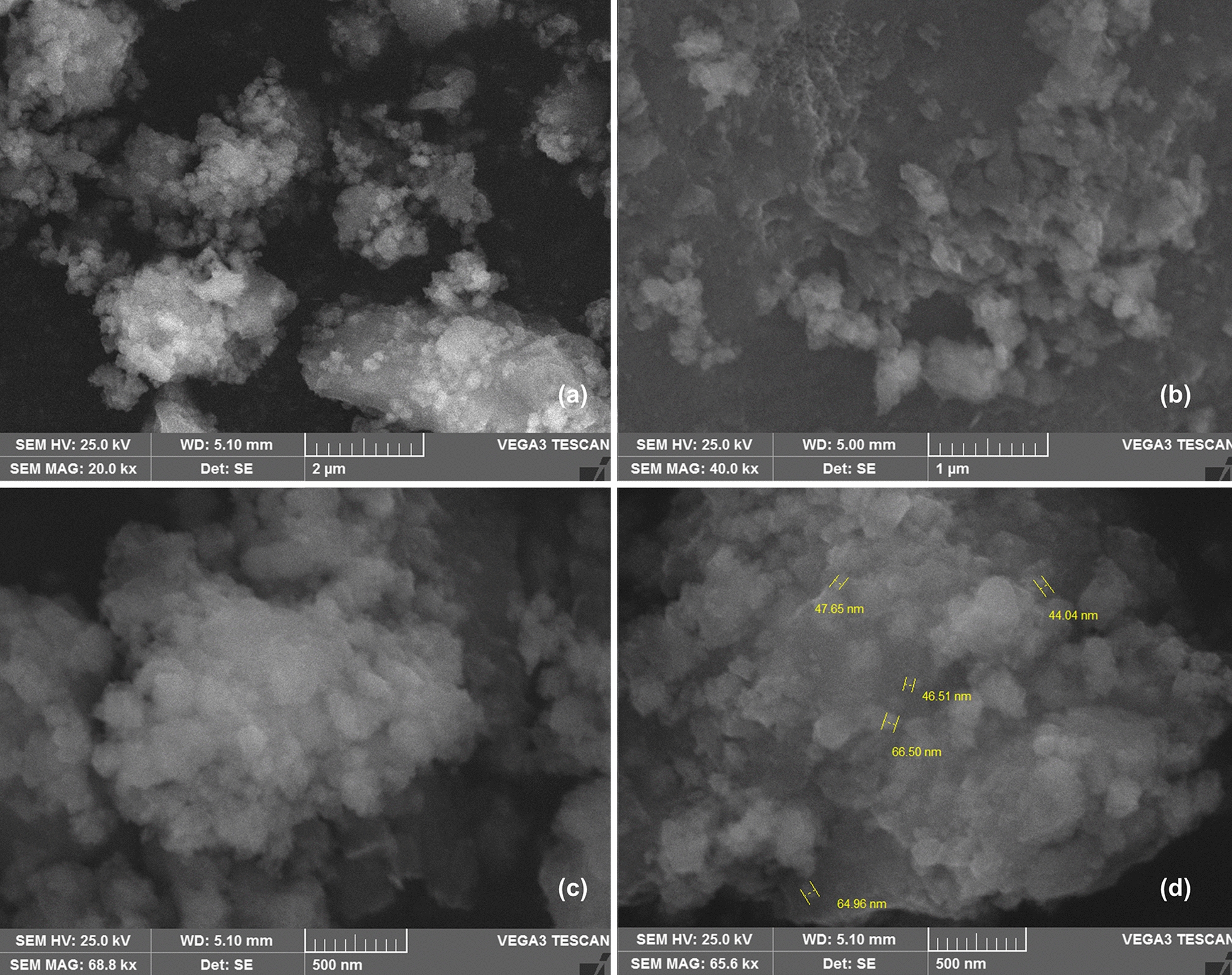
Silver nanoparticles aggregates at different resolutions **a**–**d** Under VEGA3 TESCAN SEM. observed nanoparticles (**d**) are in nanometre size range (44.04 to 66.50 nm). (Reproduced with permission from all the authors) [5]

Nano means dwarf in Greek, 1000th of micrometre (1 µm = 1000 nm). Animal cell is about 10–30 µm and protein structure is approximately 1 nm. Generally, nanoparticles are solid colloidal particles in nano size (< 100 nm) (Fig. 2) [9], and due to their exemplary size, they possess special optical and other physiochemical characteristics distinct from their powder, plate or sheet forms as they are able to confine their electrons. Their sizes can be compared with bacteria of 200 to 5000 nm (0.5 to 5 µm) in diameter and average size of 1000 nm; whereas, the subcellular bacterial vesicles are 5 to 10 nm in diameter. The largest known bacterium is *Thiomargarita* with a size of 500 µm, whereas smallest known bacterium *Mycoplasma* *genitalium* is between 200 to 300 nm in diameter.

#### Synthesis

**Fig. 3:**
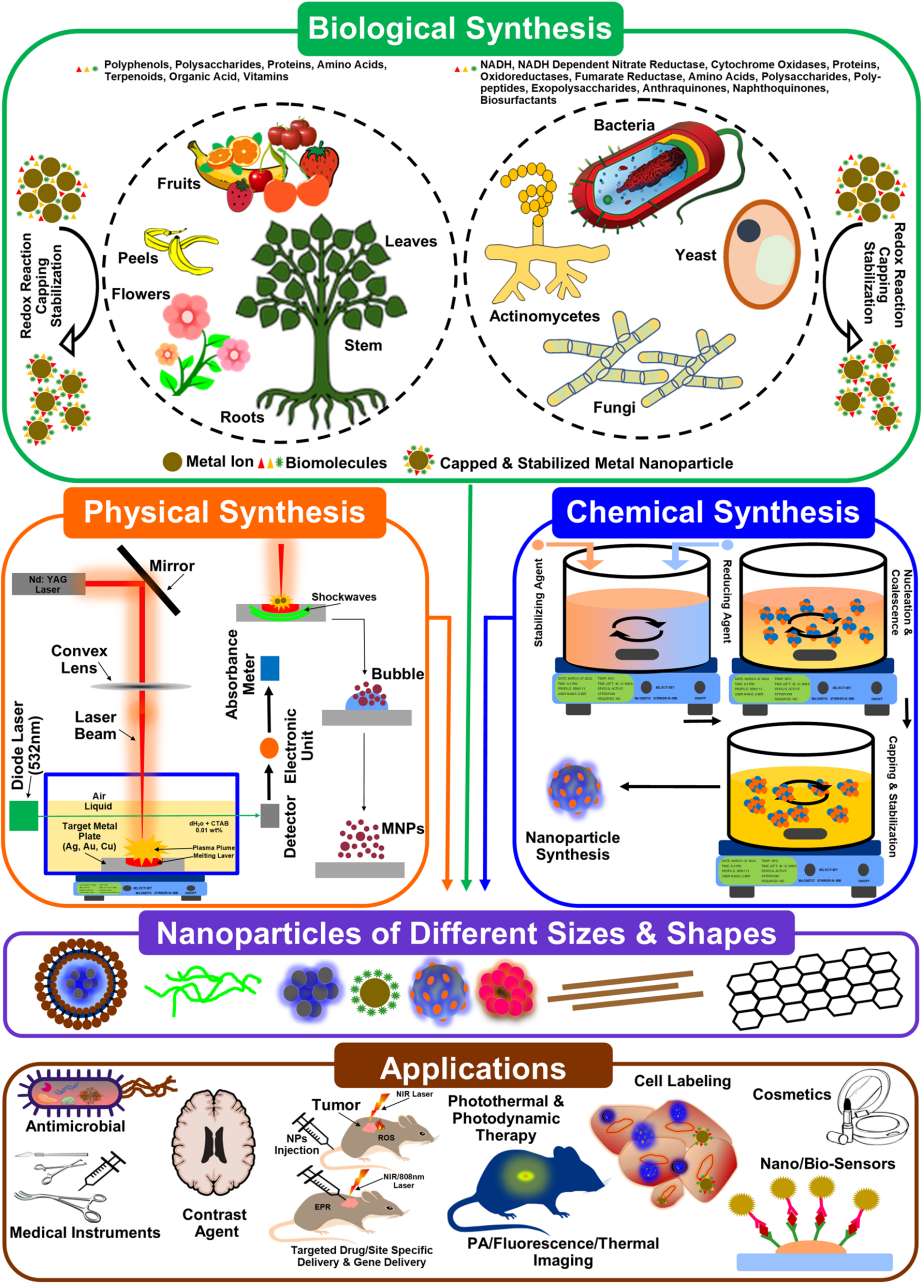
Methods of synthesis of nanoparticles. (Reproduced with permission from all the authors) [5]

Nanomaterials or nanostructures can be synthesized from inorganic (silica, quantum dots, and metal nanoparticles) or organic (liposomes, micelles, dendrimers, polymeric nanoparticles) materials through physical, chemical, or biological approaches (illustrated in Fig. 3) [5, 10, 11]. Based on the applications and biological effects, nanostructures in diverse shapes, sizes, or chemical compositions can be synthesized with the intention of conjugation with drugs of choice, controlled dispersity, target delivery, and functionalization in therapeutics [12, 13]. If functionalized with appropriate biomolecules or drugs, NPs are able to bypass the immune cells, stay in the system for longer period, higher distribution, reach target tissue in higher concentration, avoid diffusion to adjacent tissue, release therapeutic agents or drugs on specific stimuli to longer duration at a specific rate, and yield desired effect that can be used as imaging or contrast agent [4].

#### Inorganic nanoparticles

Due to their unique physio-biochemical properties like surface plasmon resonance (SPR), inorganic NPs, mainly MNPs (Fig. 2), have provided some extended applications in drug design and development; SPR is believed to be due to free electrons in outer shell of MNPs and responsible for zeta potential, biological activities, and interface with other charged surfaces or structures like biological cell [5]. Functionalized-iron oxide NPs carrying anti-cancer drug paclitaxel can yield superparamagnetic as well as receptor-mediated targeting effects [14]. Other effective inorganic NPs are nano-ceramide-GO NPs and black phosphorus nanosheet functionalized with polymer for enhanced circulation, biodistribution, superior delivery, and finally greater anti-cancer biological effects [15, 16]. Nanobiotechnological research has evolved with the development of array of inorganic NPs or small-sized particles (Ag, Au, Cu, zinc oxide, manganese oxide, cadmium oxide, molybdenum, aluminium, iron oxide or tungsten) especially for the non-conventional drug candidates, thanks to the salient features associated with their dimensions [17–22]. Due to the distinct biological characteristics, silver (Ag) as an element has found its relevance in drug development and wider application in various biological assays. [23]. Because of physical dimensions [24], odds for penetrating skin, particularly damaged or wounded skin can be seen higher in case of nano-sized elements including silver [25]. The existence of biomolecules-capped NPs improves the affinity for microbial as well as animal cells [26]; also provides antimicrobial activities in biofilm mode [11, 27–31]. Among all elements, silver is believed to be the ideal especially for the development of NPs; and, as a result of higher affinity for microbial cells, AgNPs have been seen with higher bactericidal and fungicidal activities [11, 32]. Due to that, a wider application of silver-based compounds has been seen in order to control inflammatory and microbial proliferation [33–35]. Apart from that, catheters, wound dressings, orthopaedic devices, and even dental implants have been coated with different silver nanomaterials in order to inhibit the microbial infections subordinated to them [36].

#### Organic nanoparticles

**Fig. 4:**
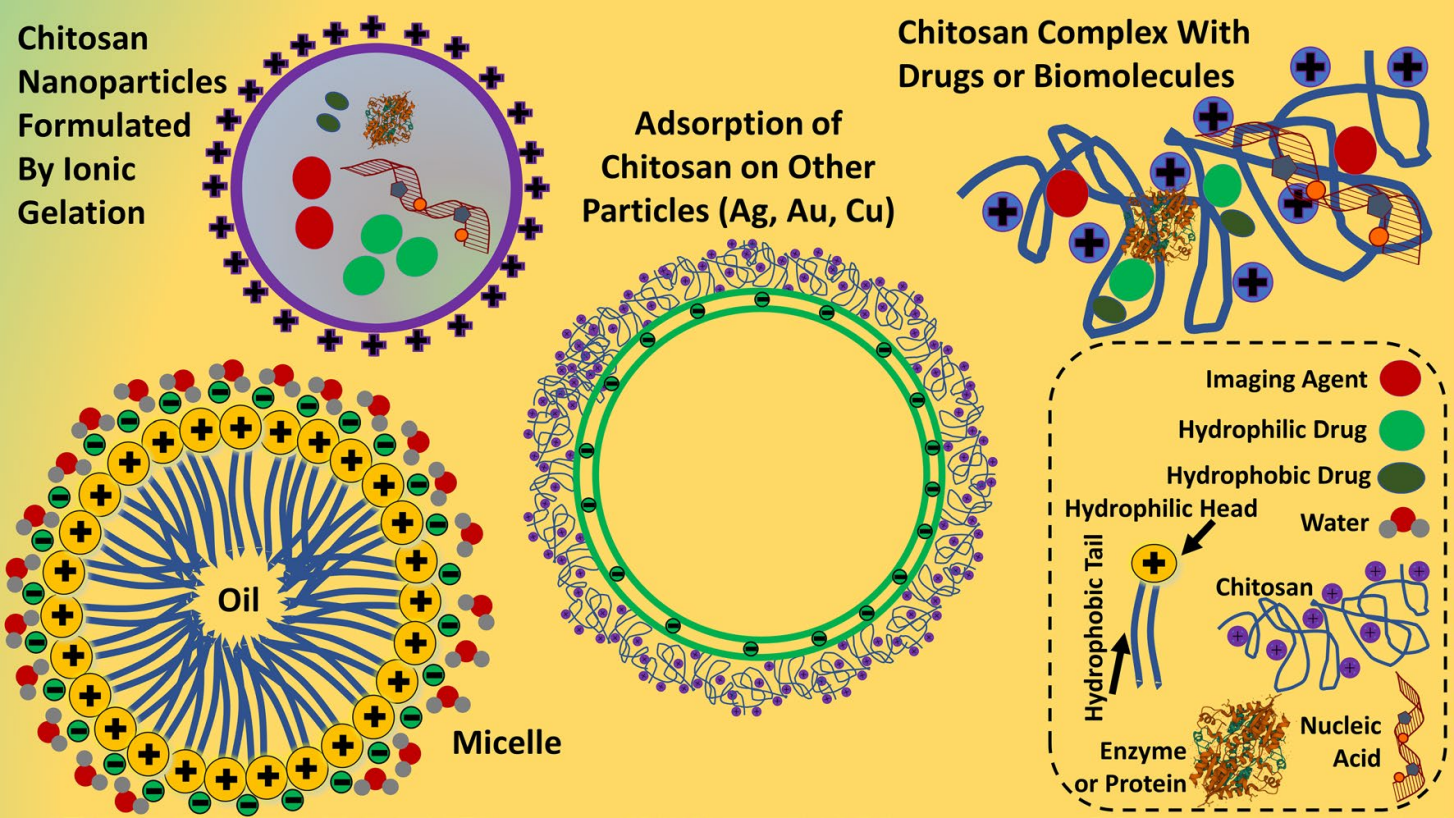
Organic nanoparticles and their complexes

**Fig. 5:**
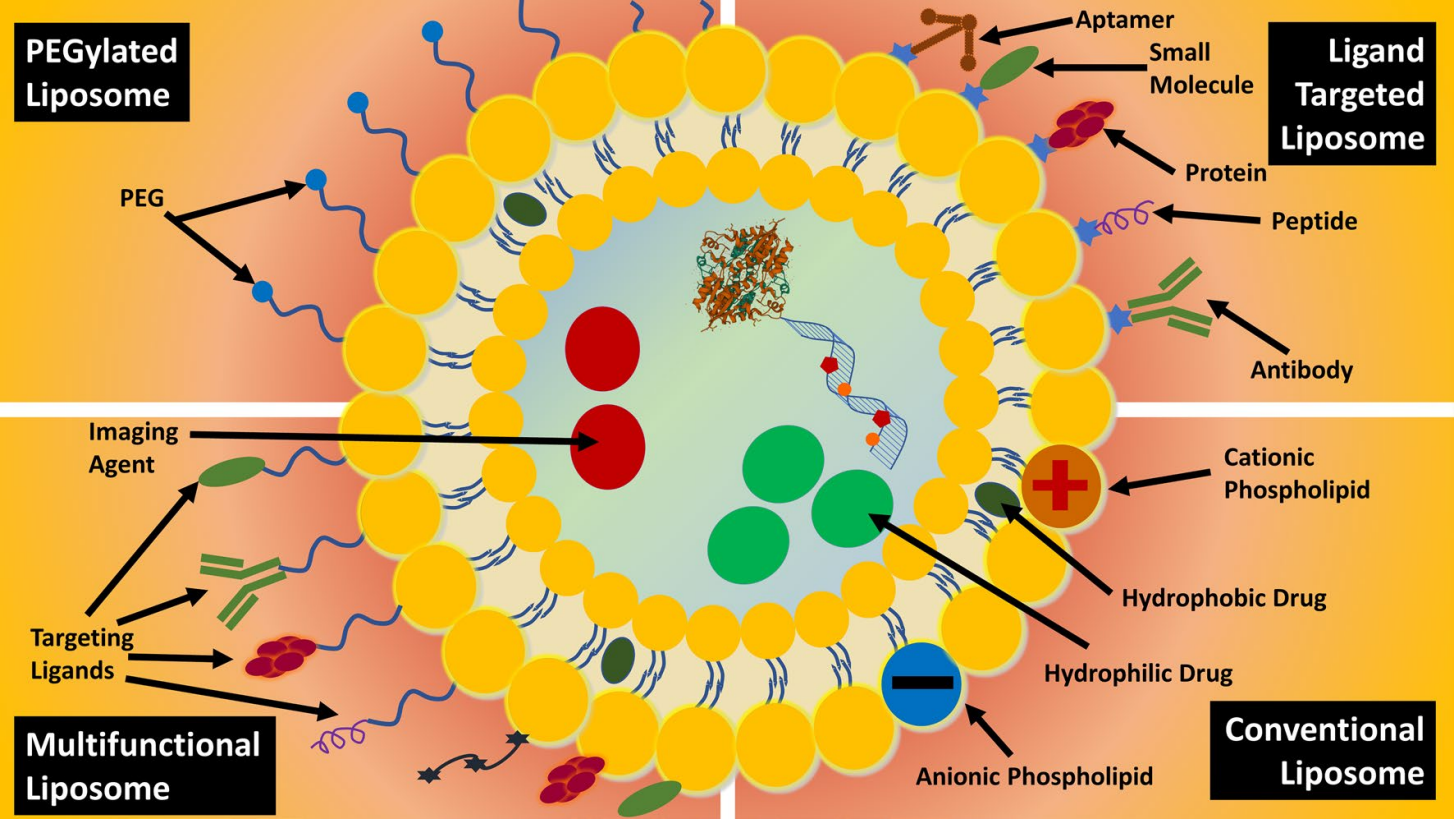
Liposomes and their functionalization with other biomolecules, drugs, or antibodies

Biocompatible, biodegradable, and versatile polymeric NPs like chitosan nanoparticles (ChNPs) (Fig. 4), PEG, PLA, PCL, and PAA NPs are equally effective, however most of their synthesis requires either multiple chemicals or solvents [37–41]. In majority of cases, NPs have been either capped with polymers or peptides (like PEGylated NPs) (Fig. 5) in order to minimize their cytotoxicity to healthy cells, also, to enhance their therapeutic effects [40, 42]. Although, such procedures require multiple steps as well as resources. Not only polymers, NPs based on lipids, liposomes (Fig. 5), and nanoliposomes have been formulated through thin-film dispersion method to encapsulate or cargo drugs like brinzolamide within stabilizer hydropropyl-beta-cyclodextrin to improve bioavailability and aqueous solubility; also, nanoliposome was prepared with mannose-cholesterol conjugation by PEG of diverse molecular weight [43, 44]. Unique structure of peptides in lipid-NPs would have properties of both polymer and liposomes; so, greater aqueous solubility, distribution, prolonged release, and pharmacokinetics can be achieved. Among organic NPs, liposomes are very unique in structure (Fig. 5) and apart from a variety of general manufacturing techniques, they can also be formulated with different sizes, composition, lipid molecules, loaded with drugs (hydrophobic drug in layer and hydrophilic in core), bioactive molecules, imaging agent, or photosensitizers [45]; additionally, they can be coated with PEG, target ligands (antibodies, peptides, proteins, or carbohydrates) (Fig. 5), or left without any surface modification or functionalization [40, 46]. Such distinctive structural features make them ideal carrier for both hydrophobic (in lipid bilayer) as well as hydrophilic drugs (in aqueous core) (Fig. 5) [47, 48]; additionally, more than two types of drugs or combinations of drugs, contrasting agents, and photosensitizers can be carried or encapsulated within one liposome (multilammelar) [46, 49]. Such freedom of carrying different biomolecules provides sequential release with dissolution of each layer.

#### Dendrimers, fullerenes, and nanobodies

**Fig. 6:**
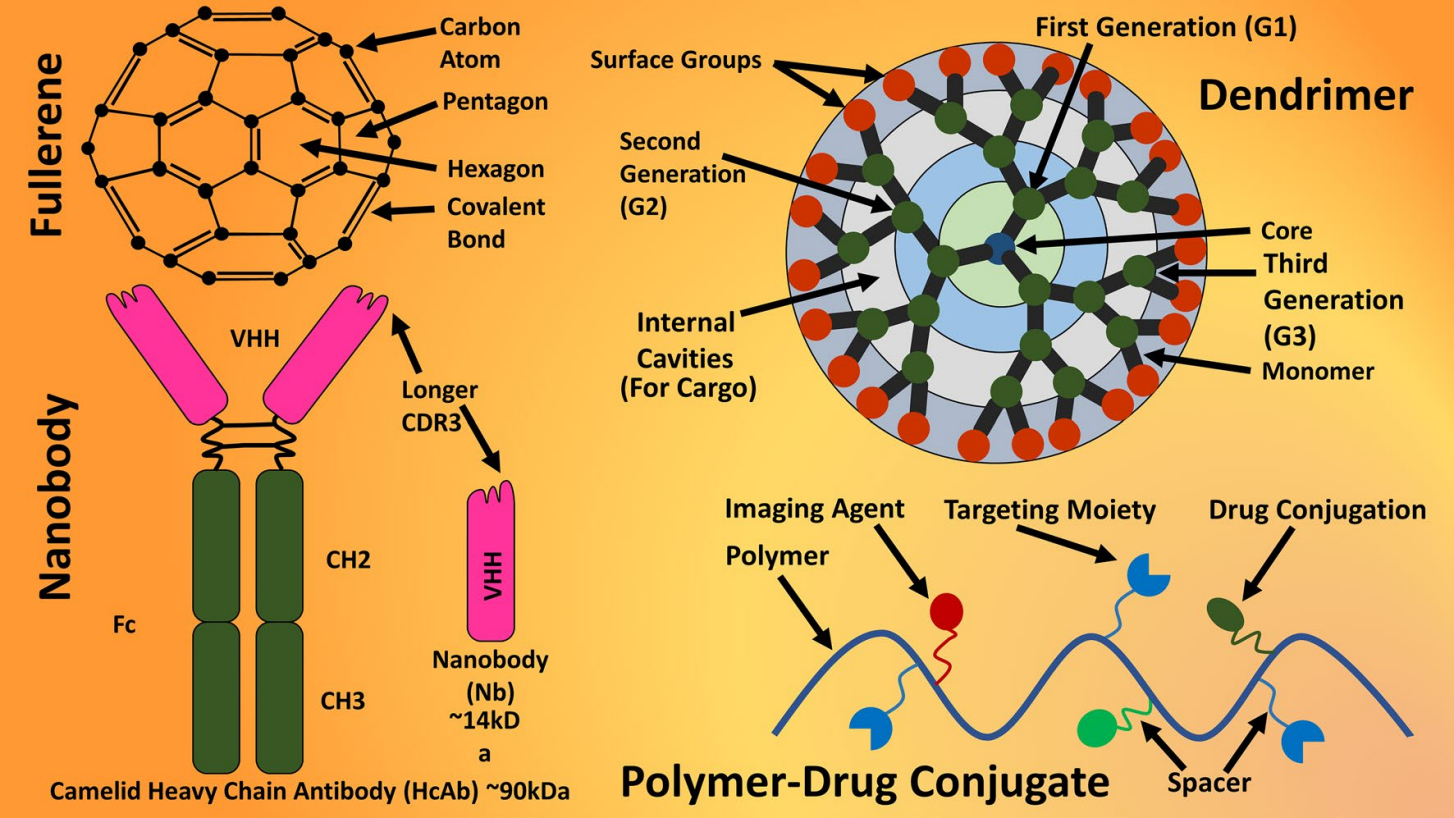
Dendrimer, fullerene, nanobody, and conjugate of polymer with drugs, imaging agent, and targeting moiety

Dendrimers (Fig. 6) are highly organized, ordered, and defined artificially synthesized polymeric macromolecules with high number of functional groups in a compact molecular structure; due to their nano structure, homogeneous, and monodisperse structure, they have been considered in drug delivery systems for cancer and imaging [50, 51]. They are hyperbranched macromolecules with end-groups protruding out of the periphery (Fig. 6) and if functionalized, the physical and biological properties of dendrimers can be modulated. Their special characteristics have made them potential nanocarrier. Internal voids inside dendrimers provide enough sites for drug conjugation and due to this, dendrimers present special pharmacokinetics features like higher cellular uptake, target delivery, circulation, and retention [52, 53]. Fullerene (Fig. 6) are allotrope of carbon and can be found as a hollow sphere, tube, or even ellipsoid. They are made up of single- or double-bonded carbon atoms in a partially or fully closed cage or mesh; and due to that, they are excellent drug delivery system with wide variety of applications like cancer.

A number of bioactive molecules and drugs can be carried and released from fullerenes in controlled way to target tissue; due to multivalency in fullerene structure, a number of drugs or biomolecules can be conjugated easily and ideal for various biological targets. One of the classical applications is the conjugation of antioxidants to fullerene and inactivation of multiple free radicals to avoid excess biological damage that could have initiated the disease progression due to uncontrolled free radicals.

Nanobodies (Fig. 6), unlike conventional antibodies, are smaller, single domain, and variable fragment of heavy-chain only recombinant antibodies [54]. They are greater soluble and highly stable, can penetrate tissue deeper and can be cleared from blood quickly [55, 56]. Just like their counterpart mAbs, nanobodies can bind to transmembrane receptors or epitopes with greater chances of novel targets, can have stronger binding than conventional antibodies; although, due to absence of Fc domain and subsequent lack of complementary toxicity, nanobodies are greater nanocarrier for therapeutic agents, toxins, peptides, or radionucleotides. As a result, nanobodies have been examined for high-quality imaging, theranostics, targeted delivery of bioactive molecules or drugs, anticancer therapy. Another kind of prominent nanocarriers are micelles (Fig. 5) with a unique structure of having both hydrophilic (cover or head) as well as hydrophobic (tail or core) parts; in its conventional setup, they are ideal for carrying hydrophobic drugs or bioactive molecules, however can also carry hydrophilic drugs if modified. Due to their cover or head, they are safe from phagocytosis and retained in the circulatory system for longer period; this is why micelles are hugely stable under physiological conditions while carrying and delivering hydrophobic therapeutic agents in its core. Hydrophobic tail of micelles can be conjugated with the biomolecules entrapped in core, thus transport of a larger amount of drug without any leakage before can be done with targeted delivery and targeting ligands. This exceptional feature of micelles makes them one of the dynamic drug delivery systems.

**Fig. 7:**
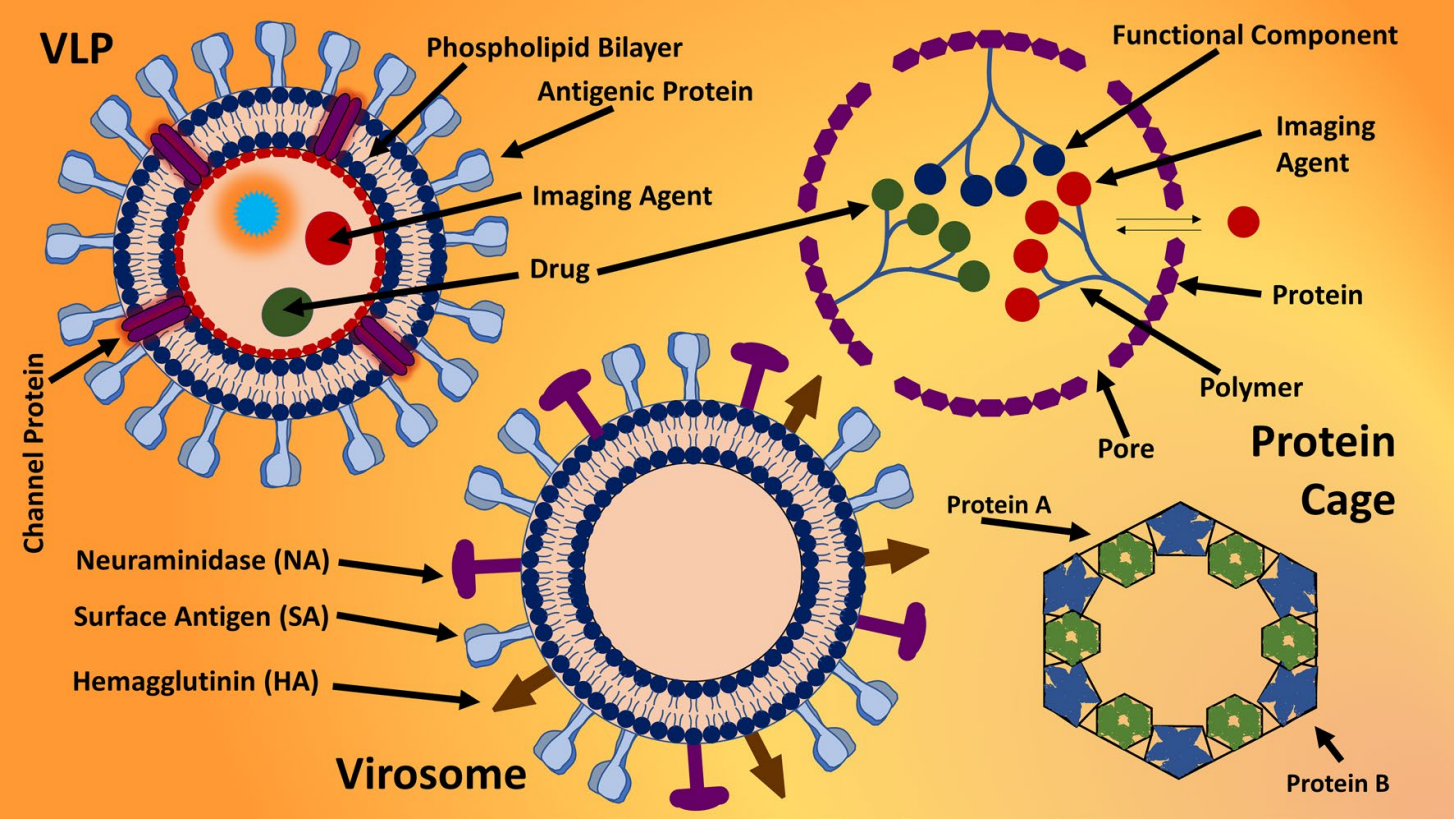
Virus-like-particle (VLP), virosome, and protein cages

Apart from organic NPs, polymers can be conjugated (Fig. 6) with greater amount of low molecular weight drugs, imaging agents, or bioactive molecules; resultant conjugate are ideal and very reliable nanocarrier with higher solubility, stability, retention, and penetration to cancer cells. Polymer-drug conjugates often present targeted and prolonged delivery of drugs for an extended period; however, conjugation with drugs can impact the molecular weight and finally the distribution of drug intracellularly. Conjugates of polymer and drugs (Fig. 6) can be designed and synthesized for triggered-release of cargo, according to the environment of disease sites like more acidic tumor area; this way, the conjugated drug cargo can be released in a controlled manner. Due to polymeric structure of conjugates, a greater bioavailability as well as biocompatibility can be achieved; this was seen with the anticancer combination therapy of paclitaxel and doxorubicin. Other effective nanocarriers in nanobiotechnology are virus-like-particles (VLP) and caged proteins (CP) (Fig. 7). VLPs are NPs with protein structure, identical to viral structure, but lacking the viral DNA/RNA; they are structurally and morphemically identical with virus-isolated structure. Whereas, CPs are also morphologically identical to viral structure but not taken from viruses and are self-assembled protein nanostructures. Just like other nanocarriers, CP and VLP are ideal effective and efficient drug-delivery systems especially against cancers due to their potential to start immune-response. Other organic NPs not derived from viruses or eukaryotic cells are self-assembled protein NPs (protein polymers) namely collagen, soy, gelatin, albumin, and elastin; however, with the help of nanobiotechnology, these protein polymers can be used as nanocarriers for carrying and delivering drugs with characteristics of polymer NPs. One of the prominent examples of protein-polymer based DDS is albumin-based NPs (Abraxane) for the delivery of paclitaxel; whereas, virus-like-protein based vaccine has been developed against HIV.

One of the prominent polymers in DDS especially in tissue regeneration and wound healing biomaterials are nanogels; they are gels but nano in size (< 100 nm) and retain the properties of gels for being non-fluid colloidal or polymer network. Gaining fluid from adjacent environment and increase in dimensions are due to their cross-linking network made up of natural or synthetic polymer (or polymers); initially, nanogels were prepared through self-assembly or aggregation of polysaccharide polymers. As compared to other DDS, nanogels provide huge benefits or convenience like ease of preparation, carrying capacity of diverse biomolecules, therapeutic agents, photosensitizers, and contrast agents, negligible efflux of cargo before target, and application routes. Although nanogels have been examined and applied in bioelectronics, DDS, biochemistry, antimicrobial therapy, and anticancer therapy among others, but their applications in vaccines, delivery of nucleic acids, and immunotherapy are most studied.

## Mechanisms of action

**Fig. 8:**
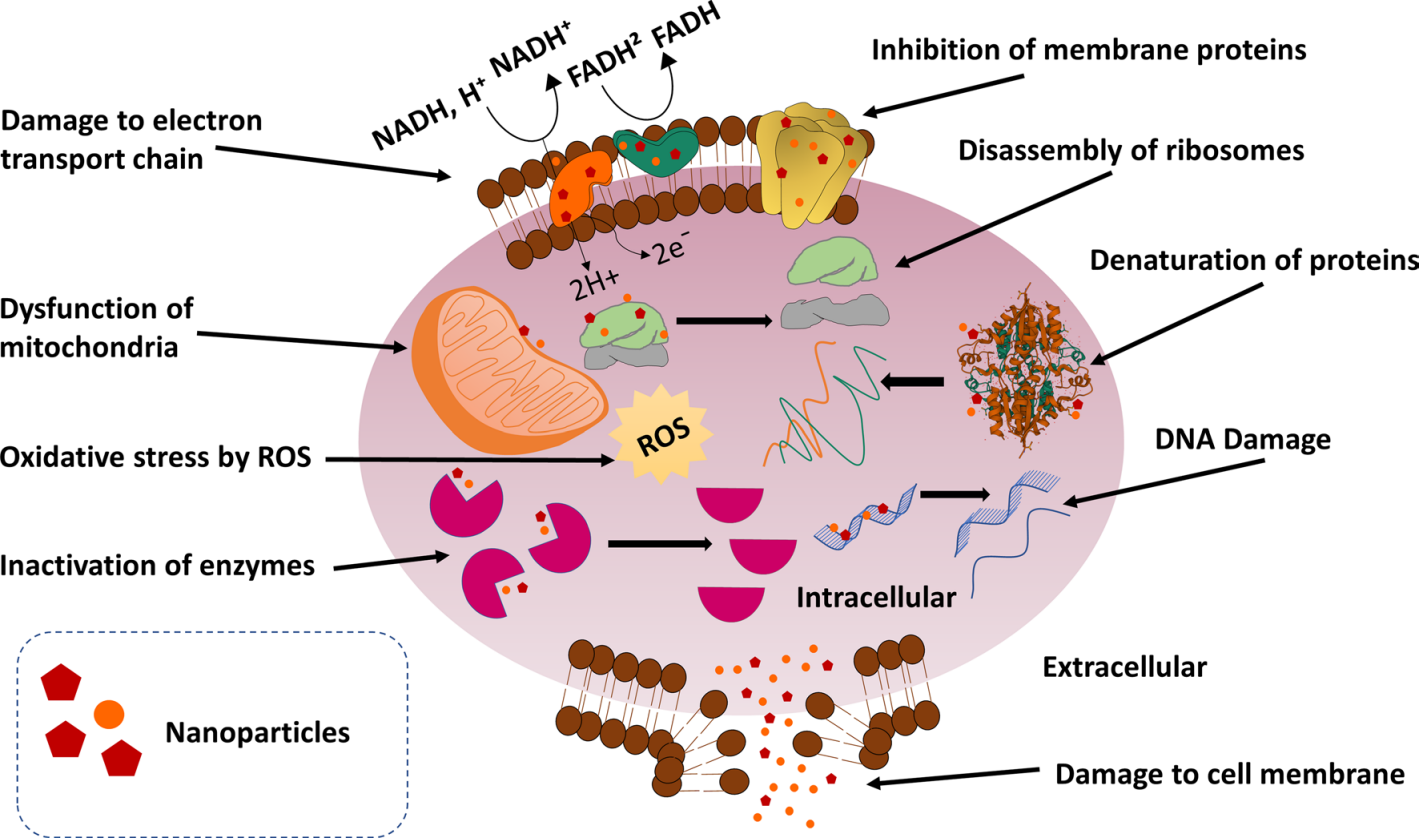
Mechanisms of action of nanoparticles. (Reproduced with permission from all the authors) [11]

**Fig. 9:**
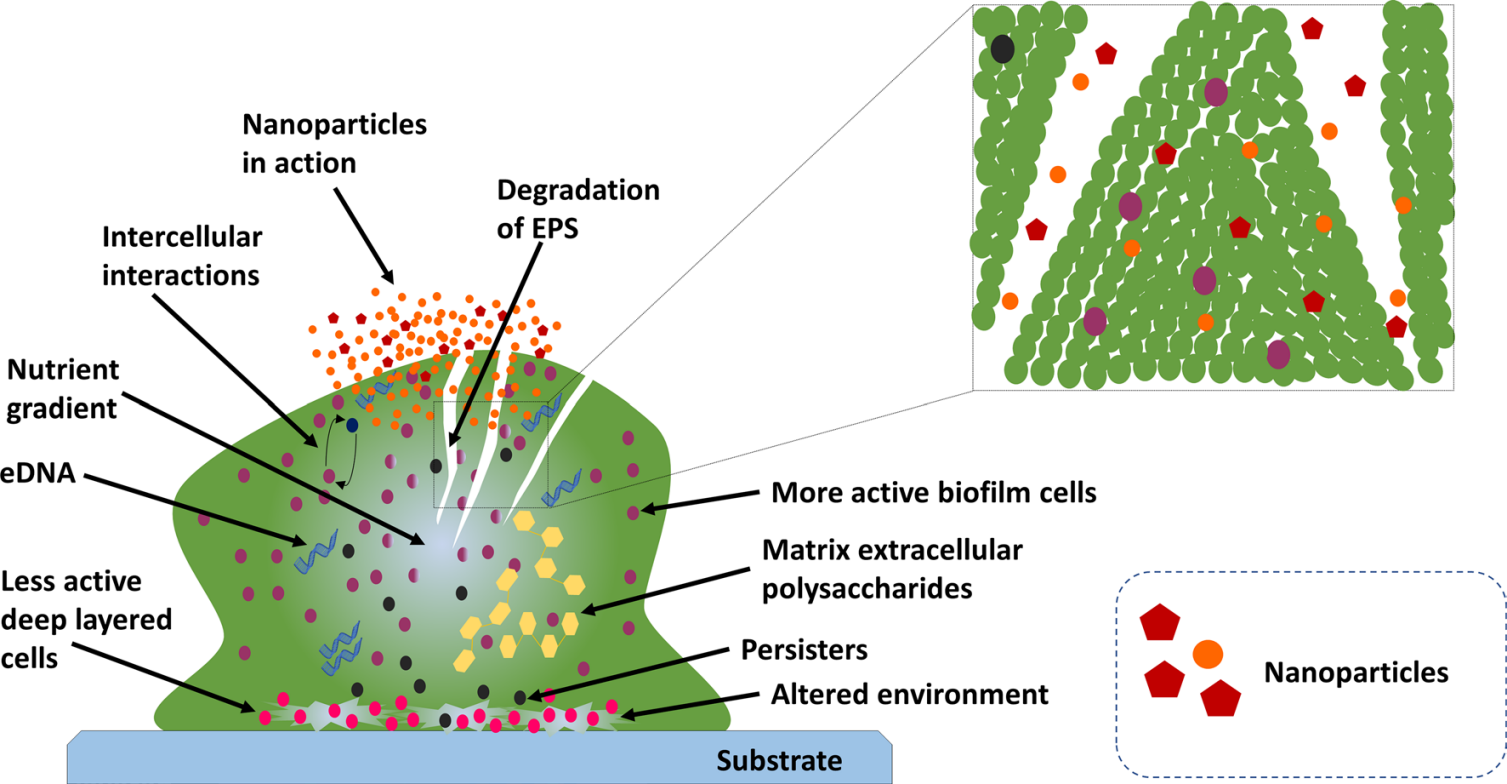
Anti-biofilm actions of nanoparticles. (Reproduced with permission from all the authors) [11]

NPs are capable of nucleic acid denaturation, to induce disorder of mitochondrial membrane potential, damaging lipids, proteins, and mitochondria (Fig. 8) by production of ROS for oxidative stress [11, 57]; and expression of cytochrome-c in order to induce apoptosis, intracellular deposition of cations, and induction of inflammation have been demonstrated [11, 58, 59]. NPs act by compromising cellular integrity [60, 61], inactivation of metabolic enzyme of transport chain by interacting with sulfhydryl group [62], and affinity for plasma membrane protein as well as phosphorus moieties of DNA in order to inactivate replication [63–65]. At the same time, displacement of Zn^2+^ and Ca^2+^ have also been suggested for biological characteristics of AgNPs [66]. NPs can disrupt biofilm framework (Fig. 9) and microbial structures [5, 11]; as leading drug carriers, NPs are competent in delivering drug to target site or tissue, can provide extended permeability and retention (EPR) effect, and induce endocytosis [35, 67–71]. In our previous article, we had explained and discussed the mechanism of action of MNPs in details [11].

## Therapeutic applications of nanobiotechnology

### Antimicrobial therapy

All sorts of NPs have been examined and tested against microbial infection including multidrug resistant bacterial (MDR) strains; polymer-based NPs, immune cell-based nanoformulations [72], and liposomes are some of the most successful NPs-based drug delivery systems applied for sustained release of conventional antibiotics without enhancing the concentration. Best example for that is ciprofloxacin-loaded liposomes (Lipoquin) for respiratory infection, capable to release antibiotic for extended period without causing any adverse effects; this way, the liposomal formulation can abolish the need of repurposing (reposition/reprofiling), high concentration, or combination. Due to its structure and characteristics (explained in Sect. "Nanoparticles"), liposomes are excellent nanocarrier for anti-fungal drugs like amphotericin B for reduced cytotoxicity; for that, it has been used for neutropenia, histoplasmosis, or even viral infections. Organic NPs like chitosan nanoparticles (ChNPs) prepared with ionic gelation method (193 to 530 nm) [73] and electrospray method (average size of 200 nm) from low molecular weight chitosan have been found to be effective against MDR pathogens like *Neisseria gonorrhoeae,* planktonic and biofilm state of oral microbes including *Staphylococcus* spp., *Enterococcus* spp., and *Candida* spp. [74]. Being the potent antimicrobial agent against MDR microorganisms [5, 11], AgNPs have been used in developing implant materials by incorporating them with polymers [75], an antifungal [76], anti-inflammatory [77], antimicrobial [5, 11], and antiviral agents [78]. In various forms, AgNPs have also been widely applied for controlling wound infection [33–35]. Some of the NPs-based methods used or applied in treating microbial infections are Silverline, Verigene, Acticoat, and Endorem. Nanocomposites of silver, fluoride, and chitosan synthesized using chemical method have also showed effective antimicrobial effects against pathogenic *Enterococcus* spp*.* and *Candida* spp.; however, nanocomposites of less than 10 nm were more toxic (mouse macrophages) than nanocomposite of more than 10 nm [79]. Polymeric NPs formed with conjugation of chitosan with microcin J25 AMPs have demonstrated dose-dependent bactericidal effects against tetracycline-resistant pathogenic *E. coli* K88 and MRSA, with no cytotoxicity to *Caenorhabditis elegans* [80, 81].

### Tissue regeneration

**Fig. 10:**
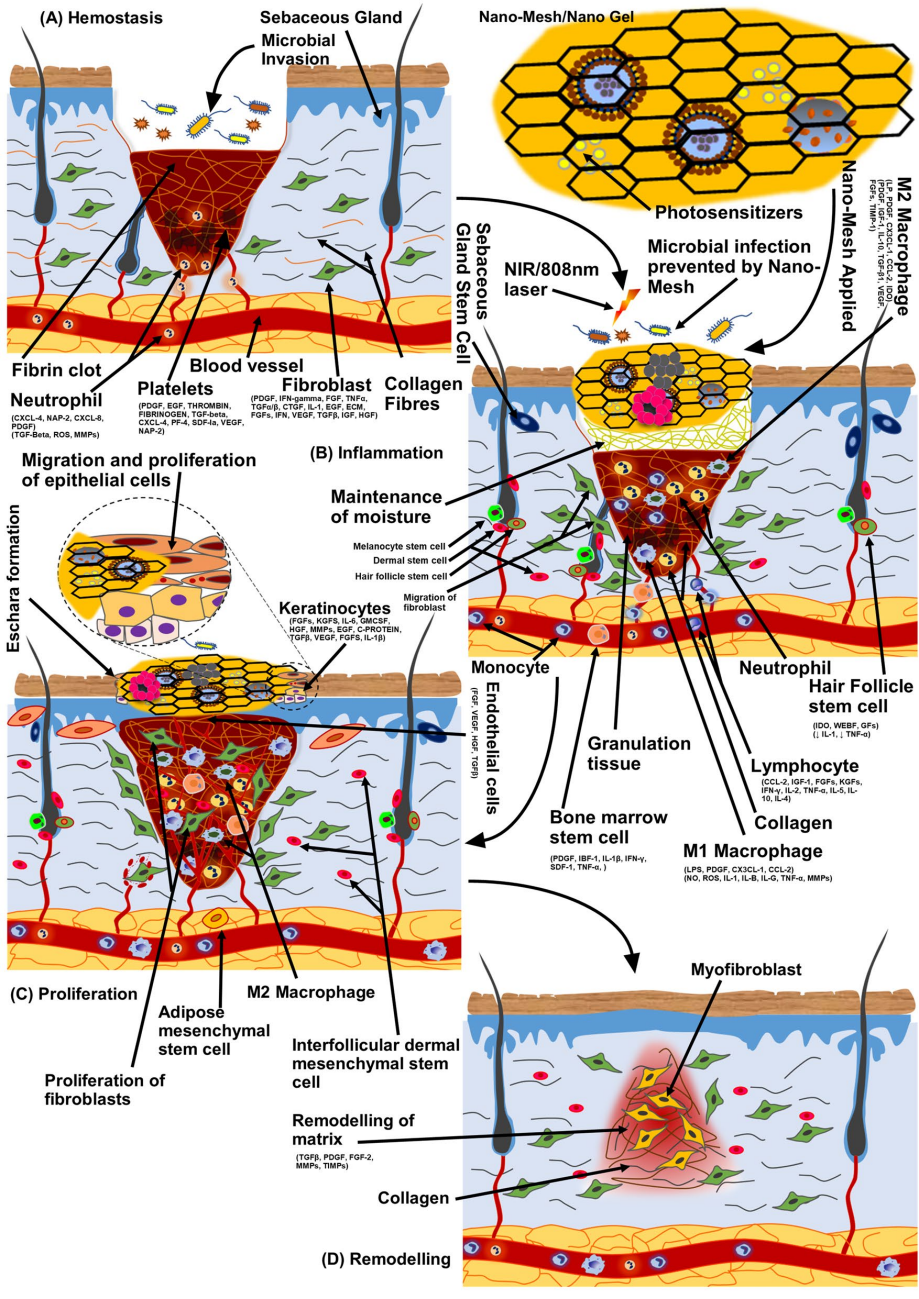
Wound healing process and application of nanobiotechnological products. (Part of the figure reproduced with permission from all the authors) [5]

**Fig. 11:**
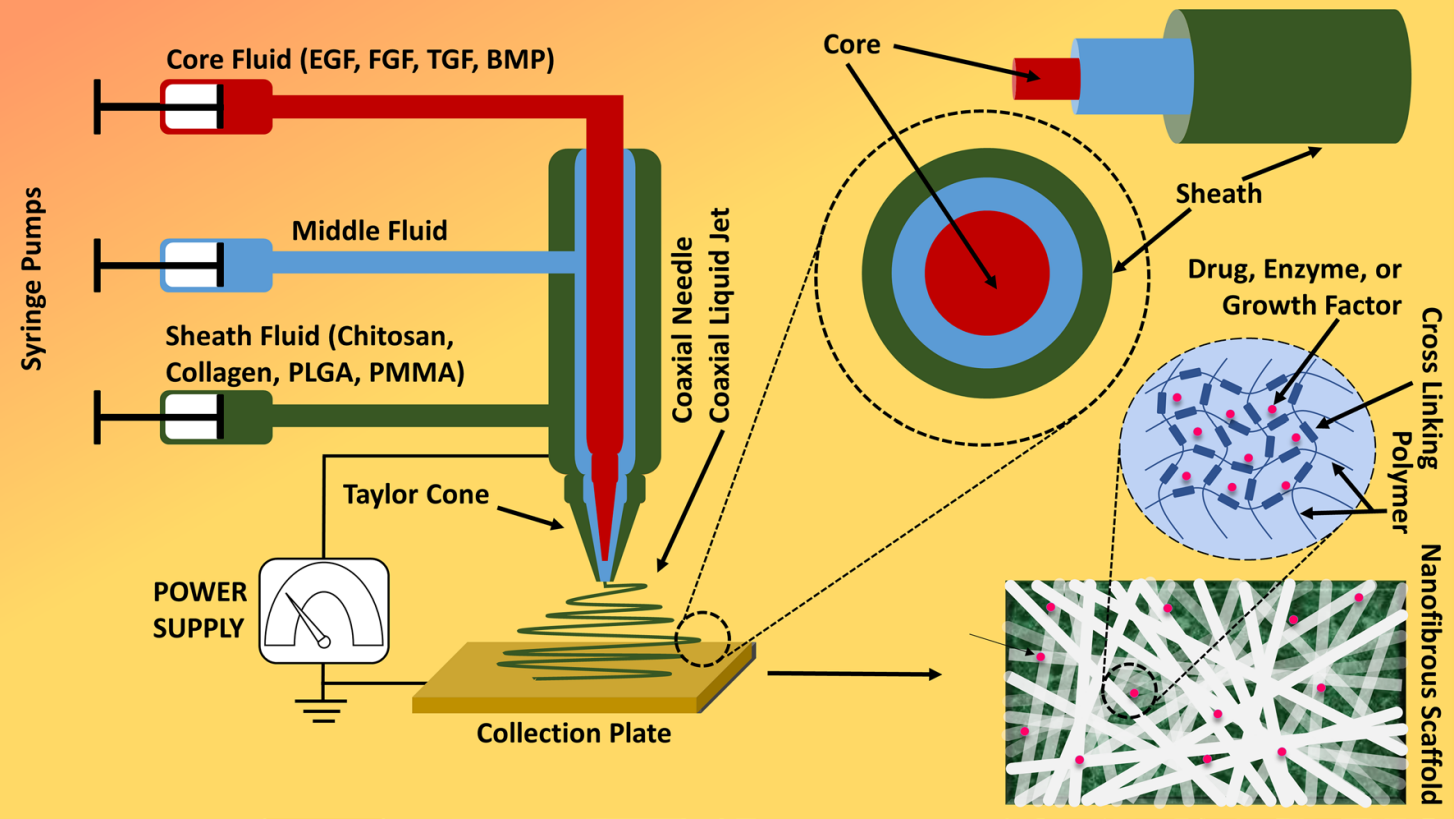
Co-axial electrospinning method. (Part of the figure reproduced with permission from all the authors) [6]

**Fig. 12:**
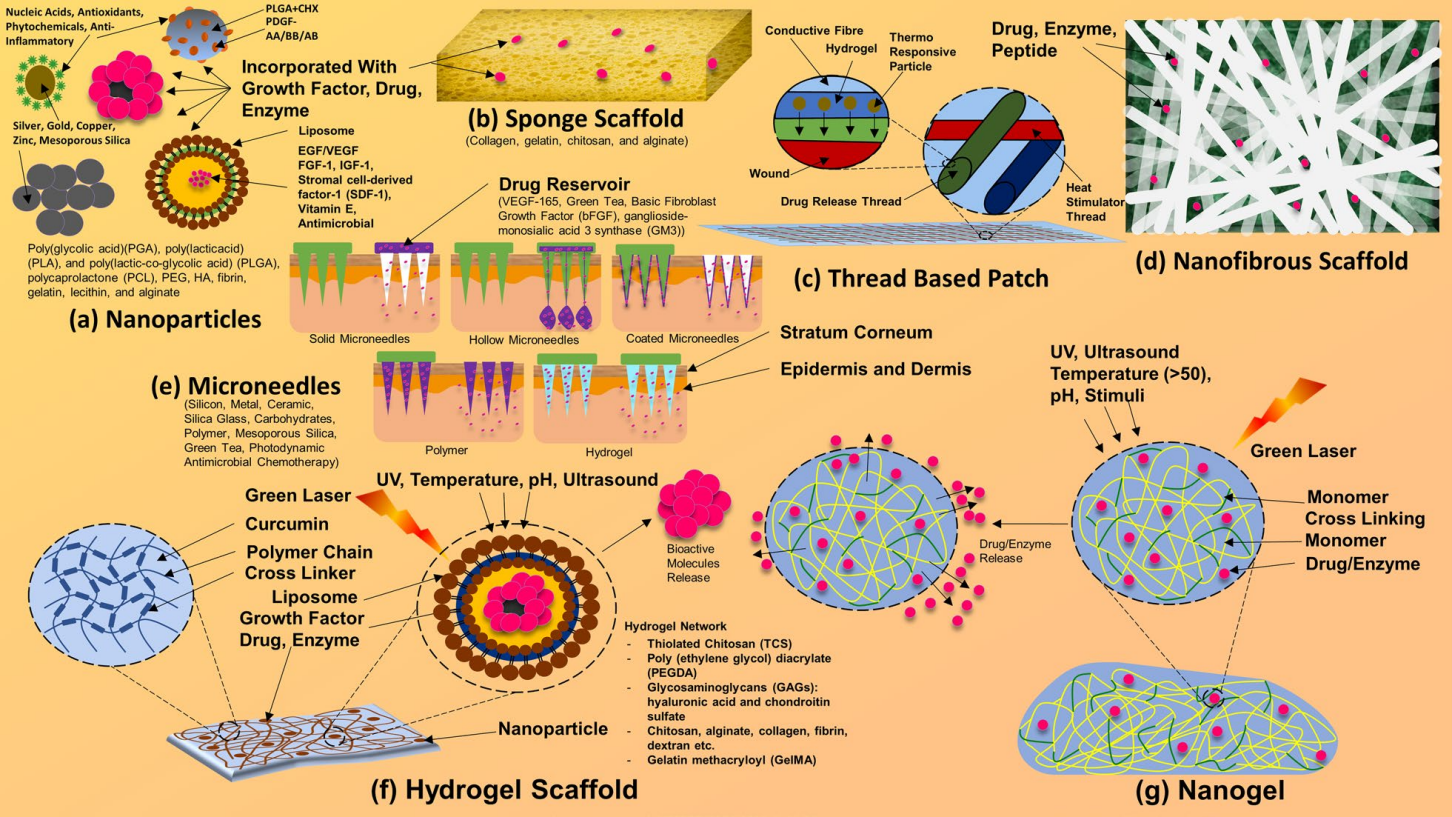
Biomaterials and nanobiotechnological products in tissue regeneration and tissue engineering. (Reproduced with permission from all the authors) [6]

Due to delayed wound healing coexisting with MDR microbial infection (Fig. 10) and immunocompromised state, scientists have shown much dedication and interest in this field of tissue engineering (TE) and regeneration (TR), tissue transplantation, and alternative biological approaches to repair or reconstruct the lost or diseased area of tissue, skin, or organ [6]. Rather than conventional bandages or drugs, functionalized-NPs can act and interact with underlying cells, environment, and microbes more efficiently [82–85]. Therefore, NPs-based biomaterials and TR methods provide an environment to speed up the TR by interacting with cells and oxygen-deprived microenvironment [86–89]. Underlying stem cells and enzymes respond to the microenvironment, initiation, and materials crucial for the TR and remodelling efforts [90]. Thereby, a number of fabrication techniques (like electrospinning and coaxial) (Fig. 11) and biomaterials have been investigated for TR [90], nanofibrous scaffolds, nanogel, hydrogel scaffolds, thread-based patches, and sponge scaffolds are few of them (Fig. 12) [6, 91]. This method uses the principles of chemistry and physics by applying electrical potential with continuous supply of biopolymer to obtain a thread or filament [91]. By an improved or modified method called coaxial electrospinning, drugs, enzymes, or cells can also be injected along with biopolymer through a common injection system. Drug or enzyme in core and biopolymer as shell or sheet of the nanofibers (Fig. 12) or filaments can be obtained through advanced coaxial system. Through electrospinning or coaxial methods, a number of fabrications of nanofibers have been applied and investigated in TR mainly for wound healing, ulcers, and lesions [92–95]. We had reviewed few of them previously. Manufactured skin-substitutes, patches, or scaffolds primarily act as barrier (like skin) and initiate faster wound healing with the help of incorporated drugs, growth factors, enzymes, or cells (fibroblasts, keratinocytes, or osteoblasts) [5].

Scaffolds or substitutes fabricated for soft tissue are different and impractical for regeneration of hard tissues like bone and dentine, also, biomaterials need to provide hardness, strength, porosity, and texture identical to hard tissues. Calcium phosphate-based hydroxyapatite (Hap) matrix is one of the widely used biomaterials or bone substitute due to its similarity with the bone matrix [96, 97]. Additionally, it is found to be biocompatible enough to induce osteoblasts to initiate bone formation when used by incorporating into nanofibers [97, 98]. On the other hand, due to lack of continuous remodelling and other physiological processes, several tissues including cartilages are hard to regain or repair post inflammation or damage, faulty presentation of progenitor cells is believed to be the main reason for this. Therefore, incorporation of either undifferentiated (MSC) or differentiated (chondrocytes) cells can be investigated for cartilage regeneration and TR methods can be exploited in this regard [99]. Nanofibrous scaffolds or cartilage matrices of different formulations incorporated with abovementioned cells have been observed with improved tissue regeneration [100, 101].

Collagen/chitosan scaffolds containing AgNPs (at 10 µg/ml) (Fig. 12) has been found to promote wound healing with anti-inflammatory effects [102]. AgNPs and their complexes are widely utilized for antimicrobial and wound healing actions [6, 103]; the incorporation of metal and silver containing compounds into gels [25, 104], hydrogel or gelling fibre [102, 105, 106], mesh or polymeric membranes (Fig. 12) mentioned as one of the effective solutions for the development of unique bandages for the wound dressings with antibacterial activity [106, 107]. Functionalized nanogel and nano-mesh containing AgNPs, growth hormone, antibiotics, and enzyme (Fig. 12) are widely examined wound dressings system for enhancing immune response [108]. AgNPs embedded in wound dressing polymers, alginate, cotton fabrics, cellulose, or chitosan promote wound healing and control MDR microbial growth [109–111].

**Fig. 13:**
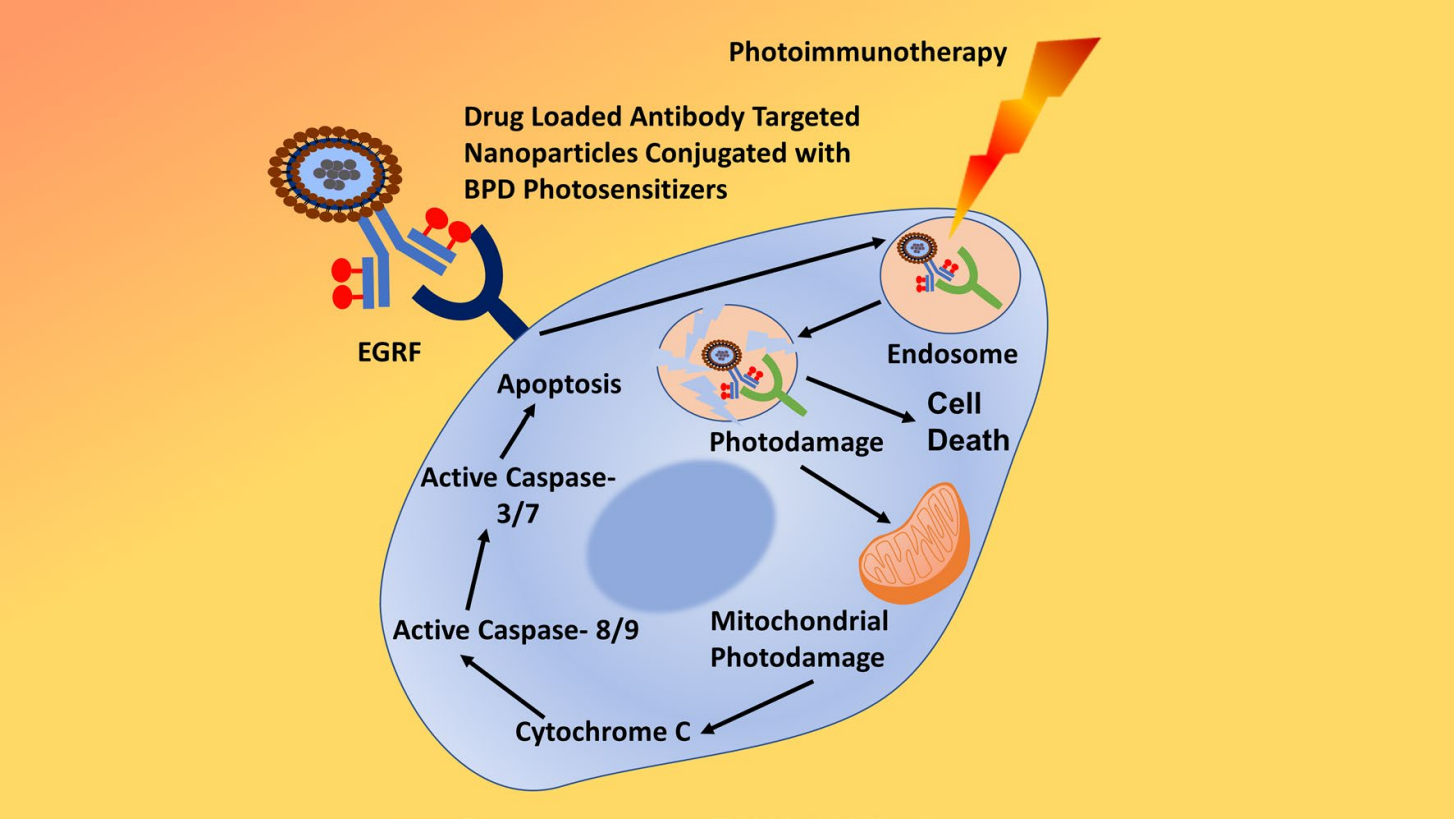
Active targeting. (Reproduced with permission from all the authors) [5]

To avoid unwanted cytotoxicity and for improving efficacy, therapeutic agents or drugs need to be delivered at the desired or target site (targeted delivery or active targeting) (Fig. 13) and this has given the concept of ‘controlled’ and ‘sustained’ release of drugs [112, 113]. Polymeric or liposomal formulations have played significant roles, in targeted delivery of enzymes [114], anticancer drugs [115, 116], and antimicrobial drugs, through ligands at a dedicated rate [112, 117, 118]. As describes previously, entrapment or encapsulation of bioactive molecules requires a cavity or casing to be avoided by reticuloendothelial system or leakage; structural features of polymeric nanocarriers serve the purpose by carrying greater quantity of enzymes, proteins, or even nucleic acids [119]. Another effective nanocarriers such as nanofibers, fabricated by a number of techniques including electrospun method, are known to have ECM like structure to incorporate or trap bioactive molecules; additionally, drugs can be embodied directly through solution or emulsion [91].

Using co-axial electrospinning (Fig. 11), nanocapsules, nanotubes, nanochannels and nanowires carrying drugs, enzymes, or bioactive molecules (Fig. 11) can also be fabricated for releasing them at target sites; also, growth factors like EGF, FGF, TGF, bone morphogenetic protein (BMP), neurokines, and neurotrophins can be delivered for wound healing or tissue regeneration [91, 120]. Under organic nanostructures, PLGA, PVA, polyamide, PMMA, and PEVA are some of the widely investigated polymers for entrapping and co-delivering diverse therapeutic drugs, enzymes, or photosensitizers [91]; due to that, organic nanoparticles mainly liposomes can deliver hydrophobic (in sheet) as well as hydrophilic (in core) drugs at the same time (Fig. 4) [118, 121]. Consequently, co-delivery of drugs or bioactive molecules of totally different purpose and structure (therapeutic agent and imaging) (Fig. 4) becomes feasible especially in the field of theranostics, for treating bacterial and viral infections, inflammation, and cancers [115, 116]. Co-delivery is very significant as adjuvant (lipophilic in nature) delivery system for cancer vaccines (Fig. 4) also for delivering bioactive molecules [118, 122].

NPs especially AgNPs are also very potent vehicle for enzymes [24], peptides, antibodies, proteins, dyes, drugs, and biomolecules [123, 124]. Biologically synthesized AgNPs by *Setaria verticillate* extracts have also been examined for carrying anti-neoplastic drugs [125]. Doxorubicin and Alendronate dual delivery using AgNPs was effective against HeLa cells with IC50 value of 27 µM [123]. Delivery of nucleic acids can be done using AgNPs [126]. AgNPs (60–80 nm) can be equipped for triggered delivery of UV-photoactivated molecules for gene silencing [124]. Such nanocarriers can be used in gene expression as well as genetic therapy (to be discussed in Sect. "Anti-cancer therapies"). *Aerva javanica* synthesized and gefitinib-conjugated AgNPs showed 50% more effectiveness against MCF-7 cancer cells as compared to gefitinib alone [127].

### Anti-cancer therapies

**Fig. 14:**
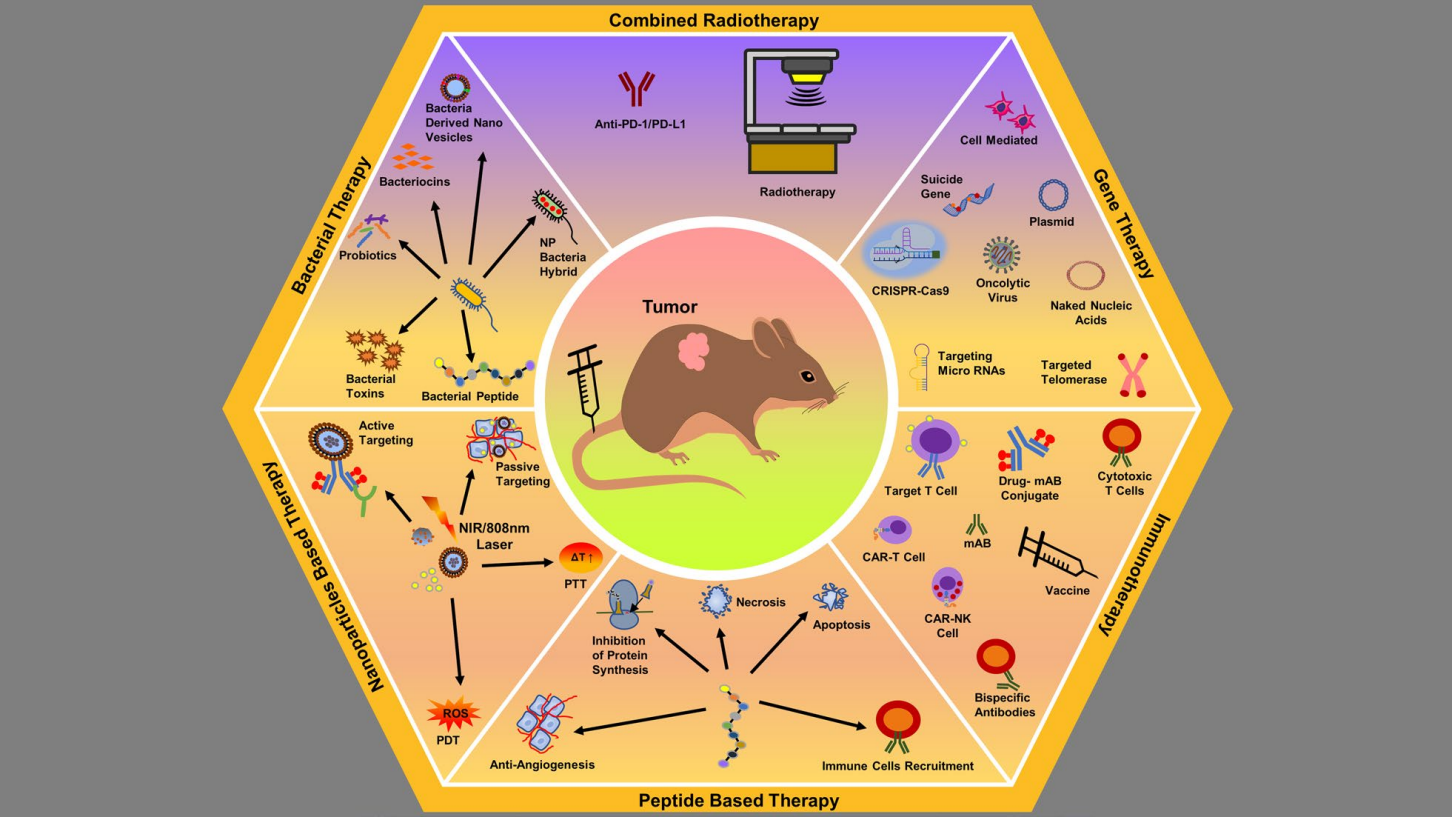
Nanobiotechnology based anti-cancer therapeutic strategies

One of the extensively examined organic NPs are PEG-PLA and PEG-PLGA based nanostructures (Figs. 4, 5, 14) for anticancer activities and used for carrying bioactive molecules or drugs (Table 1) [37, 128–130]. Whereas, due to the dimensional properties, other nanomaterials like carbon-based GO, QD, and nanotubes have also been examined for anticancer effects [131, 132]. Among the conventional or self-assembled NPs for anti-cancer effects, PEG-based NPs like PEG-platinum or PEG-Ag nanostructures have been designed and formulated precisely; such formulations have both hydrophilic as well as hydrophobic parts [133–135]. Whereas, PEG-PLA copolymer-based NPs have also been formulated and developed through co-assembly to cargo anticancer drug Capecitabine as well as hydrophobic platinum [136]. Likewise, PEG conjugated with beta-Cyclodextrin has been seen with improved carrying and delivery of anticancer drugs (Doxorubicin and Sorafenib) and multipurpose modified PEG-Cyclodextrin complex for immunotherapy as well as diabetic therapy [137] [138]. PEG-PCL (PEG conjugated with beta-Caprolactone) copolymer has been carefully formulated for delivering hydrophobic drugs or biomolecules (e.g. cytokines) against various types of cancers [139–142]. Also, PEG as shell and PAA as core as amphiphilic block copolymer assembled in aqueous solution has been seen with enhanced delivery of anticancer drug (doxorubicin) with EPR effects [143].

**Table 1:** Nanoparticles against different cancer types

Nanoparticles	Size (nm)	Zeta potential (mV)	Cancer	References
AgNPs	44.04 to 66.5	− 55.3	Breast, Colon	[5, 6]
AuNPs	14 ± 4	− 18 mV	Breast	[150]
AuNPs	49.8 ± 6.6	− 11.3	Breast	[151]
Liposomes	244.3 ± 16.7	22.9 ± 1.7	Lung, Ovarian	[152]
HSA	143.4 ± 0.7	− 5.6 ± 0.8	Breast	[153]
PTX-HAS	170.2 ± 1.4	− 17.4 ± 0.5	Breast	[153]
NHA	50.3 ± 6.2	− 21.5 ± 2.7	Breast	[154]
PLGA	210	27	Lung	[155]
PLGA	330 ± 3	− 3.9 ± 03	Colon	[155]
PLGA	204	− 5.6	Liver	[155]
PLGA	240 ± 1	− 19.5	Ovarian	[155]
SEB	389.7 ± 16.49	− 13.5 ± 12.1	Lung, Breast	[156]
PLGA-PEG-HA	265.6 ± 3.8	− 30.4 ± 0.1	Ovarian	[157]
PLGA	429.26 ± 41.53	− 11.2	Liver	[158]
LTZ-PLGA	64.0 ± 15.4	− 25.0 ± 0.4	Breast	[159]
PEG-PLA	140 ± 15	− 14 ± 4	Prostate	[129]
PEG-PLGA	114–335	− 2.8 to − 26.2	Breast	[130]
HA-GEM/CH-Pt	187	− 21	Lung	[160]
HA-CS	210	+ 25	Breast	[161]
POM-CS	105 ± 6	+ 52.0 ± 5.2	Cervical	[162]
MNP-GEM-PTX	152 ± 4	− 4.15 ± 1	Pancreatic	[163]

Another copolymer, PEG-PPG-PEG can carry anticancer hydrophobic drug retinoic acid into its micelle structure [144]; similarly, nanoparticles functionalized with polydopamine has also been designed as aptamer and formulated for efficient anticancer effects of Docetaxel as well as photodynamic therapy (PDT) [145]. On the other hand, Docetaxel has been encapsulated in PEA-based hydrophobic NPs for biological effects against lung cancers [146]. Not only PEG or PLA, other polymers mainly polysaccharides like polygalactose, hyaluronic acid, and chitosan have been used for formulation of biomaterials [128]. Natural product quercetin with anticancer properties (component of *Azadirachta indica*) can be encapsulated in chitosan-NPs for dermatological purpose for protection against UV [147], whereas chitosan polymers have been observed to inhibit intrinsic coagulation pathway. For effective PDT against cancer, Hyaluronic Acid has been self-assembled in micelles with hydrophobic photosensitizers like Chlorine-e6 [148]; such formulations have imparted enhanced and controlled delivery of cargo with greater redox-responsive kinetics [148, 149]. Apart from that, for carrying hydrophobic drugs, NPs functionalized with glycopolymers have been designed for greater triggered-release of biomolecules or drugs to cancer sites [149].

**Fig. 15:**
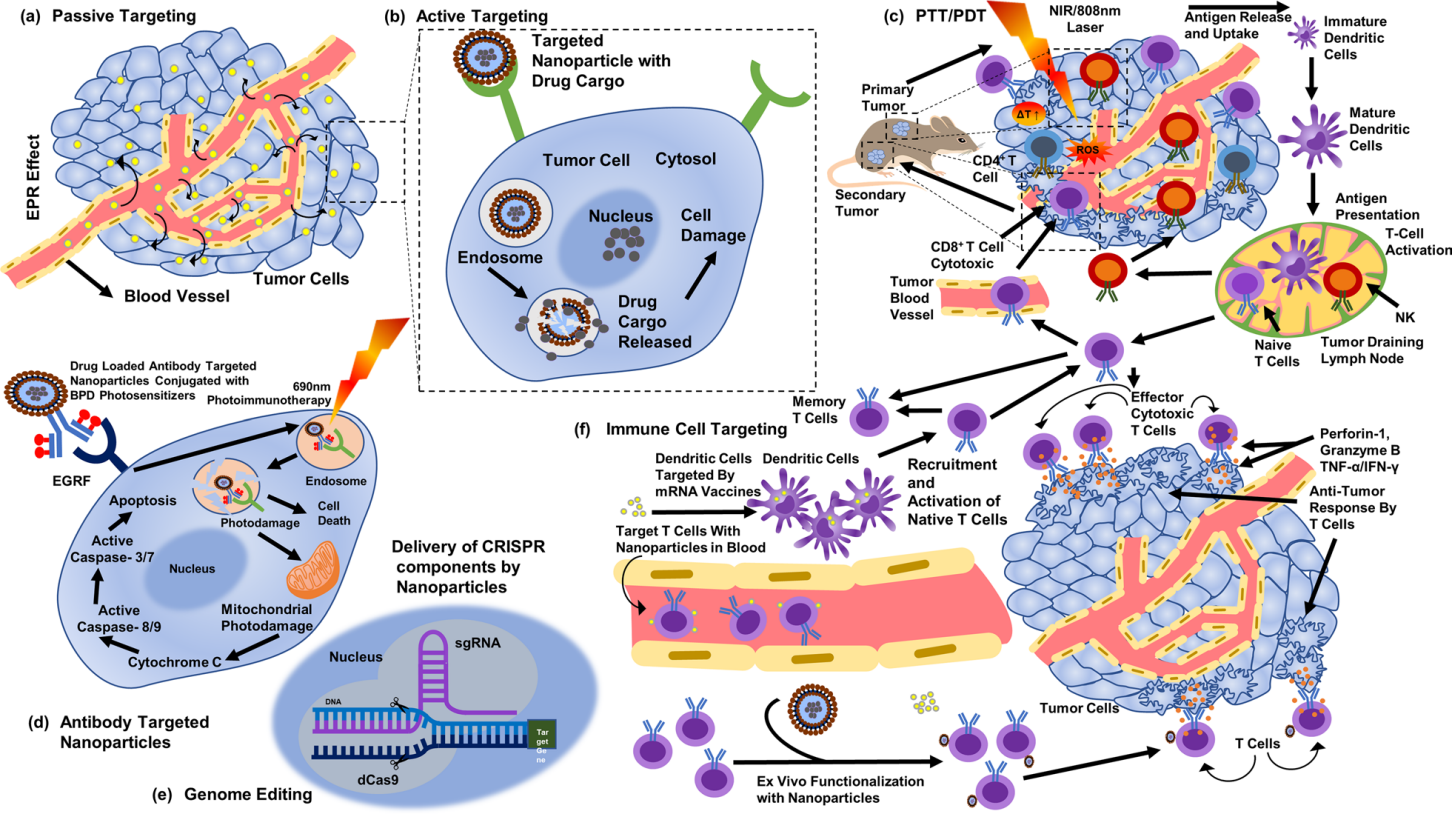
Nanobiotechnology based anti-cancer actions of nanoparticles. (Reproduced with permission from all the authors) [5, 6]

Polymeric NPs or liposomes have been extensively utilized in majority of the commercialized NP-based anticancer drugs. Doxorubicin functionalized with PEGylated liposomes (Doxil and Caelyx) was one of the foremost anticancer drugs, usually formulated by sterically stabilization of phospholipids, cholesterol, and PEG [164]. Organic NPs especially liposomes are known to bypass the reticuloendothelial system, providing more time for circulation, reduced cytotoxicity to healthy tissue, and accumulation with EPR effect at the diseased target sites due to its smaller size (< 120 nm). Apart from that, several polymeric formulations, another liposomal-based anticancer drug Vincristine-Sulfate is known for uses against lymphocytic leukaemia due to its formulation, mainly composed of sphingomyelin and cholesterol, enhance circulation time and accumulation [165, 166]. Different lipid-based and polymeric formulations are either in trial phases or have been approved for uses, like Lipoplatin, made from functionalization of Cisplatin by phosphatidylcholine (SPC-3), cholesterol, dipalmitoyl, phosphatidyl glycerol (DPPG), and methoxy-PEG-distearoyl phosphatidyl ethanolamine (mPEG2000-DSPE) [167–171]. Initial results have shown reduced cytotoxicity, therefore have been approved for treatment of rare type of cancers. Main purpose of functionalization of anticancer drug is targeted delivery to diseased or desired site also to reduce the leakage to adjacent tissues [128, 172]. Ligand or antibody targeting is one of the preferred functionalization (Figs. 4, 5, 15), increases the efficacy, like Kadcyla an antibody-targeted anti-breast cancer drug [172–174]. Kadcyla is an antibody–drug conjugate, formulated with Maytansine derivate (DM1) conjugated with Trastuzumab (Herceptin) by lysine residue of Mab, commonly target HER2 + receptor and induce cell death (apoptosis) through intrinsic pathway [173, 174]. Other nanobiotechnology-based anticancer drugs are Abraxane (albumin with NPs) and Rapamune (micelles with rapamycin) with reduced cytotoxicity to normal cells and improved anticancer effects [175].

Biogenic AgNPs (14 nm) synthesized from *Podophyllum hexandrum* Royle extracts are considered very effective against HeLa cells for inducing genotoxicity as well as caspase-3 mediated apoptosis [176]; likewise, AgNPs synthesized from *Azadirachta indica* extracts have been found to induce cytotoxicity and increased caspase-3 expression in HCT-116 human colon cancer cells [6]. As mentioned earlier, with the enhancement in physical characteristics, the biological and chemical properties of NPs can be augmented [12, 177–179]. NPs are capable to diffuse into intracellular space of cancer cells to provide EPR effect (Fig. 15) [180]; as a result, detection via cell-labelling and cytotoxicity to neoplastic cells have been seen with NPs [13, 123, 125]. Another type of MNPs, selenium NPs (SeNPs) have been observed to induce *in-vitro* cytotoxicity in oral squamous cell carcinoma (OSCC) cells and colorectal adenocarcinoma cells [181]. Recently, a number of nano-drugs like Genexol-PM® (polymeric micelle formulation), Doxil® (liposomal doxorubicin), and non-PEGylated liposomal doxorubicin Myocet™ have been authorized for anticancer therapy [182]. Recently, albumin-stabilized anti-cancer drug, Genexol-PM® (polymeric micelle formulation), Doxil® (liposomal doxorubicin), and non-PEGylated liposomal doxorubicin Myocet™ have been permitted to be prescribed [182]. Among MNPs-based DDSs, AgNPs have been examined widely as vehicle to transport or distribute enzymes [24], peptides, antibodies, proteins, dye, drug, and biomolecules [123, 124]. AgNPs synthesized through *Setaria verticillata* extracts have been examined for efficiency to cargo anti-cancer drugs [125]. AgNPs-based dual delivery of Doxorubicin and Alendronate was very effective (IC_50_ 27 µM) against HeLa cells [123]. AgNPs synthesized through *Aerva javanica* and conjugated with Gefitinib were 50% more effective against MCF-7 breast cancer cells as compared to Gefitinib alone [127]. Active targeting of cancer-cells by AgNPs-based DDS is not only feasible but also efficient [183]; additionally, distribution or supply of drug cargo in packets may also be modulated through vehicle redesign [184]. Sustained and long-term delivery (~ 30 days) of anti-cancer drugs have been seen by adjusting the basic structure of polymeric-NPs carrier [185]; such modulations have exhibited significant anti-cancer cytotoxic effects [185].

**Fig. 16:**
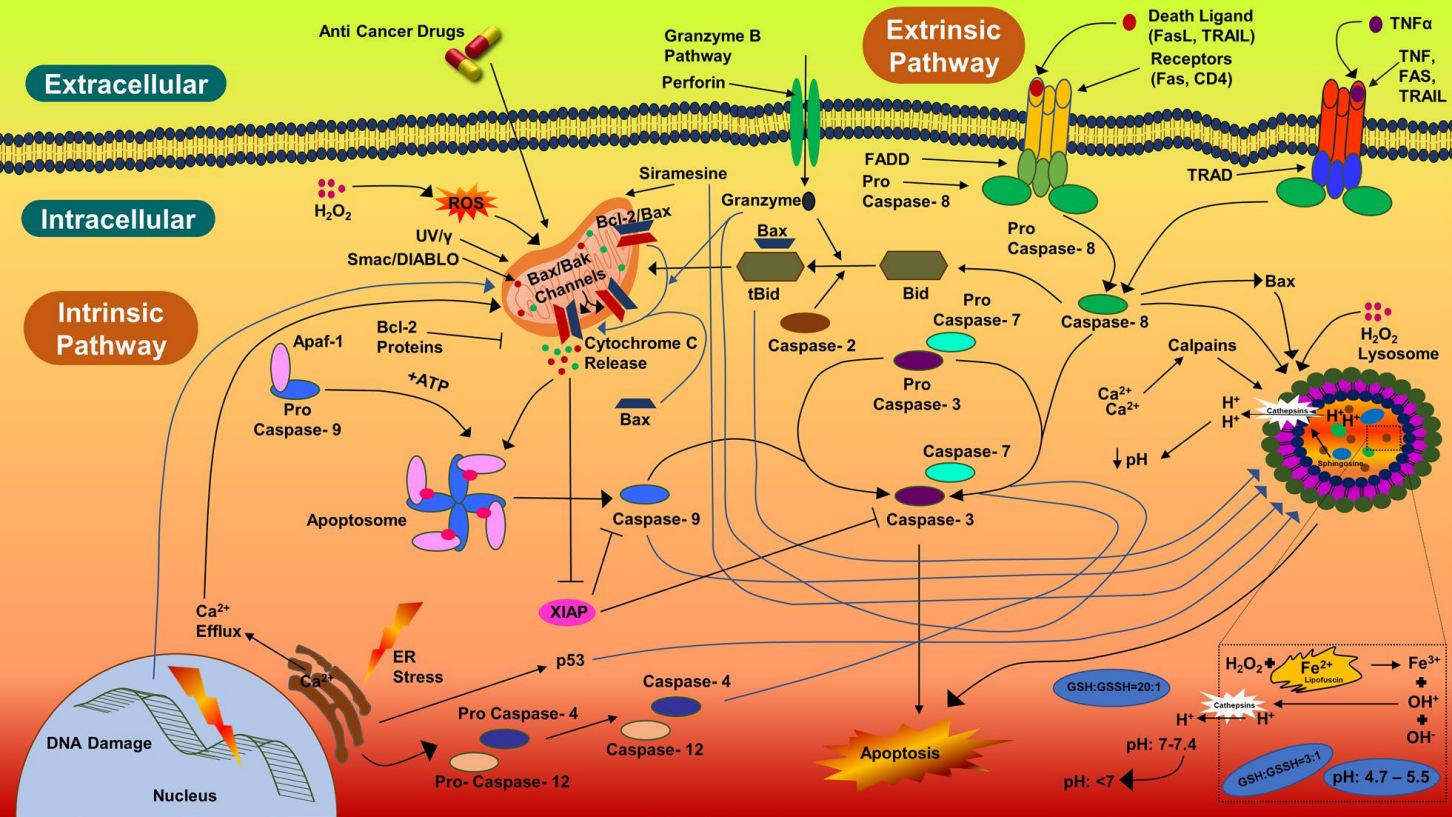
Schematic representation of apoptotic pathway. (Reproduced with permission from all the authors) [6]

**Fig. 17:**
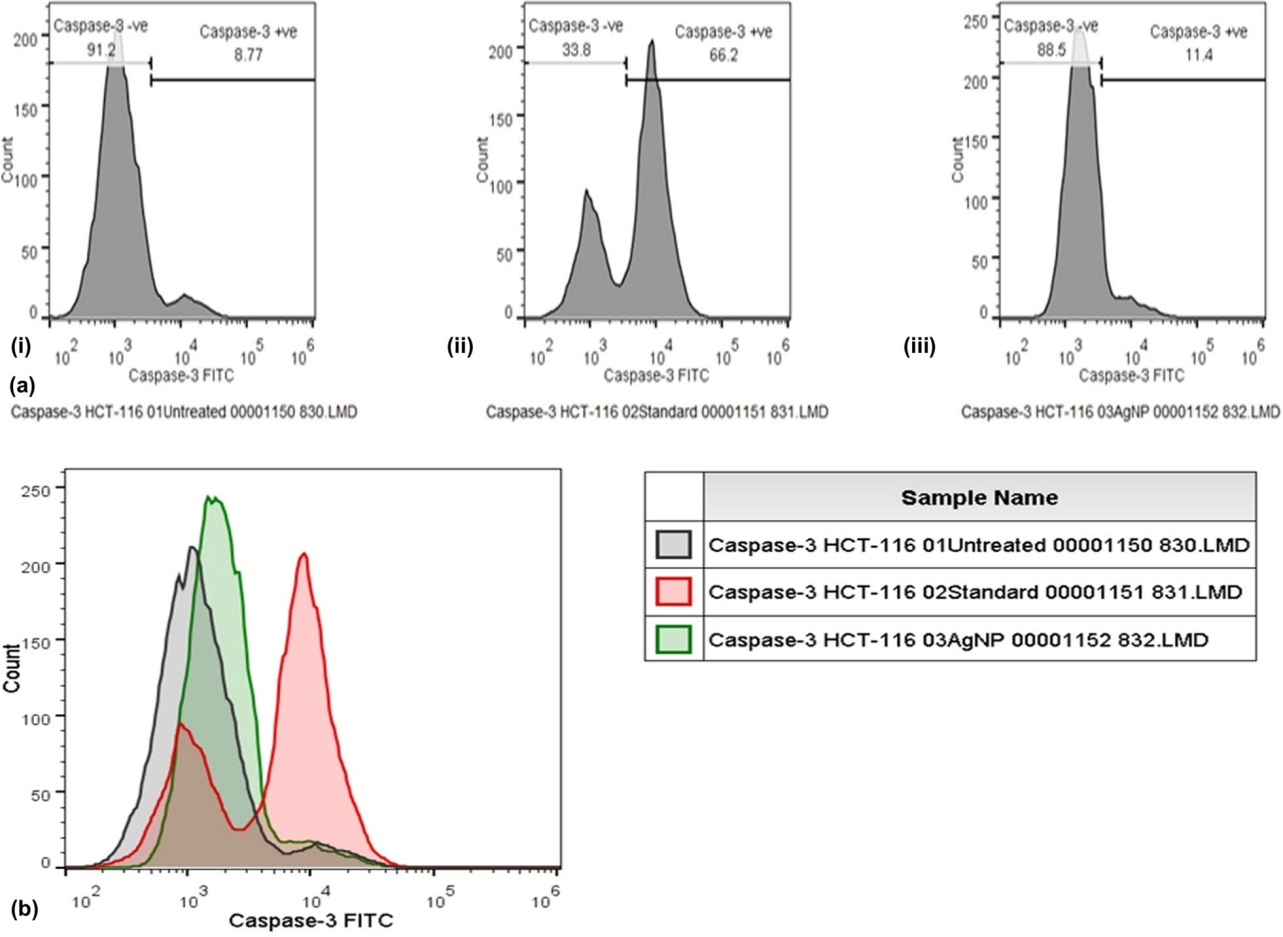
Caspase-3 expression induced by silver nanoparticles in HCT-116 colorectal cancer cells. (Reproduced with permission from all the authors) [6]

Apoptotic pathway can be prompted by MNPs, UV and gamma rays, or by oxidative stress by reactive oxygen species in order to release cytochrome-c by mitochondria and activating caspase-9 (Figs. 10, 15, 16) [5, 186–188]. Apoptotic pathway is very much likely to be initiated by like AgNPs, anti-cancer drugs, or radiation through a series of events and can be tested through quantification of caspase-3 (Fig. 17) [5]. Previously, we had investigated AgNPs (IC_50_ 744.23 µg/ml) for inducing apoptosis in HCT-116 cancerous cells and found that caspase-3 expression in AgNPs-treated HCT-116 cells were 1.5-fold higher as compared to untreated cells (Fig. 17) [6]; also, our findings in coherence with few previous studies [186, 189, 190]. AgNPs (10 µg/ml) synthesized with *Rubus fairholmianus* extract have been found to induce apoptosis through intrinsic pathway and caspase-3 expression (1.18-fold higher) in MCF-7 breast cancer cells [186]. Similarly, MNPs (12–41 nm) synthesized biogenically by *Solanum trilobatum* extracts upregulated the caspase-3 expression in MCF-7 cancerous cells [187].

Biogenic MNPs are very effective against carcinoma cells to induce apoptosis. AgNPs (53 nm) synthesized by *Beta vulgaris* L root extracts can induce higher caspase-3 activities at very lower concentrations (5 µg/ml, 20 µg/ml, and 40 µg/ml) in HuH-7 human hepatic cancerous cells as compared to CHANG normal human hepatic cells [191]. AgNPs at 40 µg/ml were able to induce elevated condensation of chromosomes and more than 26% and 24% (respectively) of cells with early and late apoptosis [191]. AgNPs (7–20 nm) at very less concentration are capable to upregulate the apoptotic activities in cancerous cell lines [192]; AgNPs were able to induce apoptosis HT-1080 and A431 at 0.78 µg/ml and 1.56 µg/ml respectively. Biogenic AgNPs (73.37 nm) synthesized from *Fagonia indica* were able to induce caspase-3 in human breast cancer cells at concentration of 12.35 μg/ml [193]. AgNPs alone can induce severe damage to intracellular structures or crucial cellular organelles by creating stressful environment. Stress or stimuli through endoplasmic reticulum damage, Ca^2+^ efflux (Fig. 16), or injury to genetic materials are usually followed by initiation of apoptotic process [194]. Therefore, it is evident that biogenic AgNPs can induce apoptosis in cancerous cells more efficiently than healthy cells by activating caspase-3 intrinsic pathway.

Immunotherapy is intended to stimulate immune cells (innate or adaptive) to identify and attack cancerous/tumorous cells (Fig. 15), whereas immunomodulation is the stimulate or supress (modulation) of immune system through natural or synthetic bioactive molecules or drugs to treat cancers or even infections [195–202]. Both of these therapeutic strategies are much effective in long-term inhibition or eradication of cancerous cells than using anticancer (cytotoxic) drugs alone to kill cancerous cells. Nanobiotechnology has provided numerous immunotherapies (Figs. 14, 15) capable to induce immune responses against cancers and microbial pathogens, like macrophage-based nanoformulations [72], monoclonal antibodies [203–205], immune checkpoint inhibitors [206, 207], non-specific immunotherapy, cytokines [208, 209], oncolytic virus therapy [210], NK Cell therapy [211–214], CAR T-cell therapy [215, 216], and cancer vaccines [217–219]. Cancer vaccines are intended to strengthen the recruitment and proliferation of native T-cell by antigen presentation to dendritic cells (DCs) (Fig. 15) [220–223]. Immunotherapies including cancer vaccines modulate (stimulate) the innate and adaptive immune systems at cellular level, whereas modulation of tumor microenvironment (TME) can cause the hindrance for CD8^+^ T cells on suppression of immune system and allowing the active targeting [221, 223]. It is already discussed that nanobiotechnology based products like nanoparticles and nanofiber scaffolds are suitable and effective for targeted drug delivery for TR, cancer, and multidrug resistant infections; adaptation of these techniques can provide impactful developments. It has been learnt that immune cells and cancer cells act both in suppression as well as progression of cancer cells [224–226], therefore the interaction between them is significant to understand the immune behaviour against cancer in order to supress or contain. Immune cells with anticancer effects include CD8 + cytotoxic T-cells, effector CD4 + T cells, natural killer cells, dendritic cells, M1 polarized macrophages, and N1 polarized neutrophils [195, 226]. Whereas, myeloid-derived suppressive cells (MDSC), tumor associated macrophages (TAM), secreted cytokines like IL-6, TNF, IL-1β, IL-23, and regulatory T cells (Tregs) are cancer inducing cells and immunoregulatory biomolecules [195]. On the other hand, depending upon the TME, cells like Th17, CD4 + , CD25 + , Foxp3 + , Tregs, and cytokines like TGF- β are considered to have dual role of promoting as well as supressing the tumor growth [223, 225, 226].

Novel anticancer techniques and methods have gained much traction in past decades. Out of them, phototherapies like PTT as well as PDT are minimum invasive methods known to exhibit potent anticancer actions with minimum systemic side effects; whereas, nanoparticles-based PTT and PDT (Figs. 10, 15) have expanded the efficacy of anticancer effects [227–233]. Apart from destruction of cancer cells with heat and reactive oxygen species (H_2_0_2_, O_3_^−^), both PTT and PDT are known to induce a number of anticancer events (Fig. 15) [6, 227, 231, 234, 235]. A number of immunological events are triggered (Fig. 15) as a result of photothermal and photodynamic destruction of cancer cells, release of antigen and presentation to DCs, release of cytokines, and activation of cytotoxic CD8 + T-cells [6, 234]; additionally, the killing efficacy of both the therapies can be improved with the help of checkpoint blockage (PD-1/PD-L1) or nanobiotechnological products like nanoparticles [206, 207, 228, 236, 237].

Apart from thermal ablation, cryoablation, ultrasound ablation, and microwave ablation are some of the newly discovered ablation techniques against cancer cells [228, 238]. Most of these methods are developed and adopted due to their minimum collateral damage and abrupt cytotoxicity to normal tissues, improved targeting, manoeuvrability, and higher killing effects [228, 239, 240]. Under novel techniques, not only primary tumor but also secondary (metastatic) tumor can be targeted, such techniques involve the direct killing effects by thermal ablation and resultant immune response; killed or destructed tumor cells initiate a cascade, antigen release, presentation to immune cells, and activation (illustrated in Fig. 15) [228, 241]. Destructed or killed tumor cells act as a source of tumor-associated antigen (Fig. 15) which then trigger the immune response against secondary tumor cells by releasing a number of cytokines and activating the anti-tumor cells (cytotoxic CD8^+^ T cells and NK cells) [6, 242, 243]. These techniques are more advanced and efficient than conventional anticancer therapies, NPs-based PTT and PDT are few of them. In addition to ablation effects and immune responses, the utilization of NPs with ablation can improve the overall anticancer effects [241, 244–246]. NPs-based PTT and PDT can provide numerous advantages, such as targeted killing, delivery of photosensitizers and therapeutic agents, functionalization [228, 247, 248]. Combined NPs-PDT and NPs-PTT can target (and kill) the cancer cells directly along with initiation of immunological cascade to target secondary and metastatic cancer cells [6, 241, 249, 250]. Such combined therapies are called photothermal immunotherapy and photodynamic immunotherapy, as they are using immunotherapy along with photodynamic and photothermal therapies [229, 249, 251, 252].

Among novel strategies in nanobiotechnology, gene therapy (Figs. 14, 15) has been developed as an independent therapeutic by deleting incorrect sequence, introducing correct gene or genetic sequence into defective or incorrect genetic sequence [253–255]. Gene therapies sometimes inducing virus in order to produce correct genetic products, treat congenital disorders, or sometimes treat viral diseases or cancers. Gene therapy can be performed through CRISPR/Cas9, plasmids, oncolytic virus, or naked nucleic acids (Figs. 4, 5, 14, 15) [254–257]. Best way to bring the normal function of gene and to correct incorrect or faulty gene is done by introducing or inserting a correct sequence or normal gene at any non-specific location or at directly replacing faulty gene through selective mutation (Fig. 14) [254, 257, 258]. Cell organelles are usually upto 10 µm, double helix DNA is 10 µm, and a typical animal cell is between 10 to 100 µm in diameter; this has provided an advantage to nanoparticles (< 100 µm) to enter these cells easily as compared to other structure. NPs are known to interact cell wall of both eukaryotic as well as prokaryotic cells in order to induce biological changes [5, 6, 11]. Therefore, NPs have been used as nano-vectors or nanocarriers for carrying genetic materials (Figs. 4, 5), viral particles, or even whole virus [259–263]. Virosome (Fig. 7) is the best example for immune-therapy, also to replace induce immunogenic reaction against cancers or bacteria. However, the same nanocarriers are potential vector for replacing faulty genes by carrying the correct genetic sequence, but with negligible or no immunogenic properties [264–266]. Therefore, to introduce correct gene or to replace or swipe faulty gene with correct gene, NPs and other nanocarriers are the potential novel subjects in area of nanobiotechnology. NPs in gene therapy are some of the most valuable nanocarrier with significant benefits, by protecting encapsulated genetic materials from degradation, targeted delivery of genes, easy access to cells by passing cellular wall, and to sustain for extended period by staying in the circulation in order to deliver genes.

### Other therapeutic uses

Nanobiotechnology has been implemented for the treatment of very prominent autoimmune as well as deadly viral diseases like rheumatoid arthritis (RA) and HIV/AIDS [267–272]. Long term effective therapeutic effects with sustained delivery have been observed with nano-Certolizumab pegol, a commonly used TNF-α inhibitor functionalized with PEG; nano-formulation can deliver anti-inflammatory drugs to the inflamed synovial membrane for upto 2 weeks [273, 274]. Apart from that, targeted delivery of NPs to the inflamed tissues (like synovial membrane) has been observed with reduced synovitis and slower bone destruction and resorption [275]. As a result of reduced efficiency of conventional anti-viral therapy (named HAART) of HIV/AIDS [276], liposomal and polymeric nano-delivery systems have been developed for delivering Efavirenz functionalized with Tuftsin; functionalized-nanocarriers are capable of targeted delivery in addition to sustained release of therapeutics, therefore minimizing the side effects on long term treatment [277, 278]. Efavirenz loaded and Tuftsin-functionalized popy(propyleneimine) dendrimers can recognize mononuclear phagocytic cells and result in significantly higher uptake by infected macrophages [278].

## Factors affecting properties and applications

### Method of synthesis of nanoparticles

Methods of synthesis can modulate and affect the surface chemistry of nanomaterials during bioreduction, can also stabilize the nanoproducts. However, the entire process is not fully controlled and may result with NPs of any size or surface chemistry, that can affect the applications. Additionally, loading or bioconjugation of drugs, enzymes, or photosensitizers might require another step (usually chemical) after biological biosynthesis as these biomolecules can be degraded easily by the phytochemicals (present in plant extracts) or microbial enzymatic actions during bioreduction. On the other hand, chemical method for synthesis of NPs is not only convenient but also versatile for functionalization and modulating the surface chemistry of nanomaterials, additionally it can be conducted at large scale. More bioactive molecules can be loaded through chemical method with accuracy and changes like addition of antibodies, targeted ligands, and photosensitizers. Biological methods are easy and do not require much resources but inherit few limitations, whereas chemical methods are more convenient and versatile but may require a number of chemicals, reagents, and resources. Chemically synthesized nanomaterials have also been observed with weak biocompatibility, but with improved anticancer actions; additionally, their antimicrobial actions are noticeable. If synthesized in a controlled standardized manner, nanomaterials produced with chemical methods can render improved biological actions; also, their therapeutic applications are higher than other nanomaterials. Unlike others, physical methods of NPs synthesis are very complicated require heavy and sophisticated machinery like NAG laser (Fig. 3); also, for functionalization, a lot of additional steps are required to be followed. Despite the perquisites of convenience in addition to well-characterized and refined product through chemical and physical methods, associated higher cost as well as the potential environmental hazards cannot be dissipated entirely [26]; shortcomings of expensive armamentariums like NAG and 532 nm laser (Fig. 3) are always attached with these methods [279].

### Functionalization of nanoparticles

The nano-bio interaction, biological fate, and targeting capacity of NPs are also dependent on the parameters of functionalization which is adding or improvement of physio-chemical properties either by addition or conjugation of biomolecules, reduction, or stabilization with different materials [12, 280]. Surface chemistry, chemical groups, or chemical composition can highly influence the antimicrobial activities, biological uptake, and cytotoxicity of NPs [12, 13]. Surface-functionalization of NPs is easily performed by addition of PEG on surface or coating of polymer, organic material like chitosan, antibodies, peptides, folic acid, biotin molecules, biomolecules from plant extracts, or deposition of NPs on such polymers (illustrated in Figs. 4, 5, 6, 7); however, the biological and physiochemical properties of NPs may vary on minor variation of surface chemistry [12, 281]. Surface functionalization has been seen improving the biological actions of NPs modified with PEG [281], polysaccharide like dextran, or oligosaccharide like chitosan [12]. Improved stability and disparity of NPs modified with mesoporous silica [12]. Starch-capped copper NPs (S-CuNPs) have been observed for inducing moderately toxicity with no morphological changes in mouse embryonic fibroblast (3T3L1) cells [282]; although, higher cytotoxicity with uncapped CuNPs comparatively highlights the significance of capping or functionalization [282].

Chitosan-coated AgNPs (Cs-AgNPs) have shown significant toxicity as well as changes in cellular morphology to RAW264.7 macrophages at 10 µg/ml [60]. It was found that selenium-NPs (SeNPs) functionalized with PLL were highly cytotoxic also genotoxicity to TR146, HaCaT, and Caco-2 cells as compared to PAA- and PVP-coated SeNPs with no toxicity to *E. coli*, *S. aureus*, and *S. cerevisiae* BY4741 [181]. On the other hand, biogenic polyvinyl pyrrolidone‐coated (PVP) AgNPs (10 to 30 nm, average size 20.5 nm) synthesized with extracellular *Lysinibacillus boronitolerans* supernatant exhibited significant antimicrobial effects against microorganism like *Fusarium graminearum,* also synergistic effect in combination with norfloxacin; but significant cytotoxicity for 2C12 skeletal muscle cell at concentration of 4 to 15 µg/ml with IC50 5.45 µg/ml*.* It is evident that functionalization can modulate or affect biological properties extensively.

### Host environment

Temperature, pH, oxygen, biochemistry, and presence of diverse pathogens or toxins can affect the biological properties and fate of NPs. Hypoxia (lack of oxygen) is a major hinderance in tissue regeneration (Fig. 12) and anticancer actions [283, 284]; also, DDS can also affect the availability of oxygen and moisture. Absence of moisture may result in the dryness, cellular death, chronic wound, and epithelialization over wound dressing material [285]. These factors restrict the applications of conventional DDS for wound healing but can be addressed with nanobiotechnological-based novel DDS for sustained delivery of drug by maintaining moisture and oxygen to the wound bed.

## Complications and challenges

### Cytotoxicity

Due to extremely small size (< 100 nm), nanomaterials can affect intracellular biochemical processes by interacting with biological structures like cell wall, organelles, and nucleic acids [5, 6]; this is one of the main concerns that can affect the normal healthy cells. In-vivo applications of nanomaterials have been seen as a matter of concern due to their physiobiological properties and ability to induce chemical changes in in-vivo cellular microenvironment; additionally, the end state of these nanobiotechnological products including NPs of different sizes and materials is not fully known. Recently, fullerene (4.7–9.5 nm) functionalized with hydroxyl group were investigated for cytotoxicity against human umbilical vein endothelial cells and found to induce cytotoxicity and morphological changes in a dose-dependent manner; additionally, on longer exposure (> 7 days), endothelial cells were unable to attach with delayed cellular growth. Such observations are significant to notice as hydroxyl-functionalized fullerenes were considered as neuroprotective if cytotoxicity to normal human cells are also found; same type of nanomaterials induce both beneficial as well as toxic effects or responses in biological systems.

NPs have been seen with a dose-dependent cytotoxicity for non-cancerous cells [286, 287]. AgNPs biosynthesized by *Streptomyces sp. NH28* biomass exhibited low viability (82.9 ± 7.5%) in mammalian cells at 25 µg/ml (IC_50_ 64.5 μg/ml) [288]. Starch stabilized AgNPs (20 nm) induced decline in viability of murine cells at 10 μM [31]. Starch-capped AgNPs induced genotoxicity in human lung fibroblasts cells IMR-90, although, the cells were unaffected beyond 100 μg/ml [289]. A significant toxicity in murine hepatocytes had been observed due to commercial AgNPs (15 nm and 100 nm, 5 to 50 µg/ml) as compared to NPs of manganese oxide, molybdenum, aluminium, iron oxide, or tungsten [290]. A dose dependent inhibition was also observed with significant cytotoxicity as well as changes in cellular morphology in RAW264.7 macrophages due to Cs-AgNPs [60]. Recently, we had found biogenic AgNPs biocompatible comparatively [5], but dose-dependent and cell-dependent toxicity have been noticed in recent years [289, 291, 292]; also, AgNPs have been observed for causing in-vitro cytotoxicity considerably in a number of cell types [292]. Polymer-functionalized AgNPs had also been seen for causing significant amount of toxicity in non-cancerous IMR-90 and U251 cell lines [289]. It is very much evident that biological actions and behaviour of nanoparticles are established on array of factors like functionalization, materials used in fabrication, physical parameters, or the drug delivery method [291, 293].

Starch-capped AgNPs exerted significant genotoxicity in human lung fibroblasts cells IMR-90 [289]. As mentioned previously, functionalization of NPs can affect the cytotoxicity as well as antibacterial properties; as, it was found that SeNPs functionalized with PLL are highly cytotoxic and genotoxic to TR146 (SCC), HaCaT, and Caco-2 cells as compared to PAA- and PVP-coated SeNPs with no toxicity to *E. coli*, *S. aureus*, and *S. cerevisiae* BY4741 [181]. On the other hand, biogenic polyvinyl pyrrolidone‐coated AgNPs (PVP-AgNPs) (10 to 30 nm, average size 20.5 nm) synthesized with extracellular *Lysinibacillus boronitolerans* supernatant exhibited significant cytotoxicity in 2C12 skeletal muscle cell at concentration of 4 to 15 µg/ml with IC50 5.45 µg/ml*.*

### Functionalization

As discussed in earlier Sect. "Functionalization of nanoparticles", biochemical interaction of nanomaterials including NPs with cell or cellular structures is very subjective to surface chemistry and availability of targeting ligands or antibodies on surface, presence of these ligands or biomolecules before target site are dependent on synthesis, type of biomaterials, and local microenvironment; therefore, modulating the surface properties becomes a challenge to keep the cargo until delivery site and protection from enzymatic degradation or reticuloendothelial system. Polymeric or organic nanomaterials and other nanobiotechnological products are easily surface-modified with antibodies, peptides, or other small molecules, they can easily encapsulate or carry therapeutic agents or photosensitizers; however, inorganic nanomaterials are not easily modified, also their carrying capacity is limited. Most of the inorganic NPs can only be surface-modified either chemically or biologically, however due to their own fixed size and solid structure, inorganic nanomaterials can’t carry drugs in their core; therefore, inorganic nanomaterials can only carry biomolecules or drug (on surface) if they are encapsulated with polymers like PEG or chitosan (e.g. PEGylated NPs) (Fig. 5). Also, any number of biomolecules can’t be attached on surface of NPs due to interaction among them, only limited kind and number of biomolecules can be conjugated on surface; however, a number of therapeutic or imaging agents can be loaded in core of liposomes at the same time (Fig. 4).

### Delivery and targeting

Methods of synthesis, functionalization, local environment, and mode of administration can influence the delivery and targeting of therapeutic agents, photosensitizers, and ligands. NPs can reach target site passively (without targeting ligands) or actively (with targeting ligands or antibodies specific to cell) (Figs. 10, 15). NPs without any surface-functionalized targeted-ligands or antibodies (Fig. 4) are more prone to diffuse into healthy tissues without reaching target site or without attaching to target cells; as a result, untargeted NPs may induce unwanted cytotoxicity, compromising the therapeutic purpose. Delivery of therapeutic agents or anticancer drugs to the diseased site becomes a challenge if cargo is released or leaked before target site or destroyed by the reticuloendothelial system, this is more common with unprotective cargo or cargo without proper conjugation; polymeric nanomaterials, liposomes, or micelles are less prone to suffer from faulty delivery due to well defined and structural encapsulation. Despite conjugation of targeting ligands, delivery as well as targeting is not entirely guaranteed due to numerous factors of TME and intracellular biochemistry with no definitive features; results from one experiment involving one particular cell type or animal model can’t be reflected or replicated exactly with other cell types or animal models. Consequently, thorough investigations are required to ascertain delivery capacity and biochemical interactions of drug with cellular structures.

### Physical dimensions

The size of NPs can highly influence the cytotoxicity in mammalian cells [79, 294]. Biological interactions of NPs with cellular structures are dependent on their physical characteristics [177]; size dependent plasmon absorption is due to the size-dependent dielectric function of the NPs and any shift or fluctuation of plasmon band would affect the absorption bandwidth [32]. However, the parameters like geometry, size, or topographical features of NPs can also affect the plasmonic resonance [177, 179]. Although there are a number of confounding factors like local environment, presence of targeting ligands, and type of nanomaterial, but the biological actions of nanomaterials are heavily influenced by the physical parameters and dimensions of NPs [179]; and characteristics like SPR can modulate or affect the biological fate of NPs [295]. It is well known that zeta potential (ZP) corresponds to the stability and longevity of NPs in a medium, ZP of ± 25 mV or higher indicates NPs with improved stability for longer period [5]; weaker ZP would impact the stability of NPs due to interparticle attraction, ZP of less than ± 25 meV would aggregate altogether in a medium to cause higher cytotoxicity [296].

## Conclusions

Nanobiotechnology has enabled the efficient delivery of therapeutic agents, such as drugs, growth factors, and genes, for cancer treatment, microbial infection, or repair of the diseased tissues, with the help of nanoparticles, nanotopological scaffolds, polymeric scaffolds, nanocarriers, or combination of them. We have observed that, for the controlled, triggered and effective delivery of bioactive molecules and loading and compartment of the cargo molecules, the physicochemical properties of the carrier materials can be controlled at the molecular level through nanobiotechnology based nanoscale tailoring approach. Features and characteristics of nanocarriers can be improved, manipulated, modified, and enhanced, with higher the capabilities and efficiencies, through capping, encapsulation, or functionalization. Focus must be on the research and development of novel methods, techniques, nano-biomaterials, and devices. Apart from few inorganic nanostructures, polymer-based novel nanostructures and nanocarriers can provide structural versatility, biodegradability, bioavailability, and biocompatibility; and with a number of ways, such nanostructures can be designed and fabricated efficiently with the help of nanobiotechnological methods and techniques.

Developed nanocarriers, especially polymeric and biodegradables, offer an immense space for further enhancements, due to their versatile structure and characteristics. Cargo capacity of these carriers can be further improved, by modifying their structure, for delivering greater amounts of therapeutic agents. Polymeric nanocarriers can also be designed and developed, for delivering multiple cargo, such as anticancer drug and imaging agent, with greater precision. Targeted delivery and precision delivery can be achieved with existing nanocarriers, through conjugation of targeting moieties. Nanocarriers, such as liposomes, can be further improved by incorporating other polymers or by changing the proportion, for delivering phytochemicals. Existing polymeric or organic nanocarriers can be improved further, for carrying and triggered delivery of phytochemicals with unfavourable solubility but greater therapeutic properties. Polymeric nanocarriers are some of the exclusive options presently available, with higher capacity of improvements and enhancements. Conventional therapeutic methods have numerous inherited limitations, but nanobiotechnological products, such as nanocarriers, can be handled, enhanced, and modified, despite having few drawbacks and limitations. One of the utmost goals of nanobiotechnological research are to explore existing methods and techniques and identify the ones with higher capacity for improvements and enhancements.

## Future perspective

Nanobiotechnology, despite having few challenges and drawbacks, offers immense opportunities that can be harnessed in delivering quality therapeutics with precision and prediction; by exploring its branched domains (like immunotherapy or gene therapy) more rigorously, bottlenecks and obstacles can also be addressed and resolved in return. By viewing the current trends, it is clear that nanobiotechnology is progressing towards multi-directions at very fast pace, with cutting-edge research at universities, laboratories, and industries. It can also be expected that nanobiotechnology would deliver novel methods, techniques, and materials to provide more reliable, sensitive, and efficient tools and analytical systems for theranostics. It can also be seen that nanomaterials and other nanobiotechnological products offer numerous potential applications, but attention must be focused only to the ones capable of improving efficiency, scientific methods and understanding, and quality of life; also, understanding for the interactions of nanomaterials with biological systems, organelles, ecology, and animals must be developed further.

## Data Availability

Yes.

## References

[1] Feynman R (2018). There’s plenty of room at the bottom.

[2] Drexler KE. Engines of creation. Anchor books, 1986.

[3] Qi B, Wang C, Ding J, Tao W. Editorial: applications of nanobiotechnology in pharmacology. Front Pharmacol. 2019; 10. 10.3389/fphar.2019.01451 PMC6904356 31866866

[4] Jain KK, Moo-Young M (2011). 1.45—Nanobiotechnology. Comprehensive biotechnology.

[5] Dutt Y, Pandey RP, Dutt M, Gupta A, Vibhuti A, Raj VS, Chang C-M (2022). Synthesis and biological characterization of phyto-fabricated silver nanoparticles from Azadirachta Indica. J Biomed Nanotech.

[6] Dutt Y, Pandey RP, Dutt M, Gupta A, Arpana V, Raj VS, Chang C-M, Priyadarshini A (2022). Silver nanoparticles phyto-fabricated through Azadirachta Indica : anti-cancer, apoptotic, and wound healing properties. Antibiotics.

[7] Kaur K, Thombre R, Ghosh S, Webster TJ (2021). Chapter 1—Nanobiotechnology: methods, applications, and future prospects. Nanobiotechnology.

[8] Dash DK, Panik RK, Sahu AK, Tripathi V, Dash DK, Panik RK, Sahu AK, Tripathi V. Role of nanobiotechnology in drug discovery, development and molecular diagnostic; IntechOpen, 2020; ISBN 978-1-78985-978-2.

[9] Neel EAA, Bozec L, Perez RA, Kim H-W, Knowles JC (2015). Nanotechnology in dentistry: prevention, diagnosis, and therapy. Int J Nanomed.

[10] Moeinzadeh S, Jabbari E, Bhushan B (2017). Nanoparticles and their applications. Springer handbook of nanotechnology.

[11] Dutt Y, Dhiman R, Singh T, Vibhuti A, Gupta A, Pandey RP, Raj VS, Chang C-M, Priyadarshini A (2022). The association between biofilm formation and antimicrobial resistance with possible ingenious bio-remedial approaches. Antibiotics.

[12] Sanità G, Carrese B, Lamberti A. Nanoparticle surface functionalization: how to improve biocompatibility and cellular internalization. Front Mol Biosci. 2020;7. 10.3389/fmolb.2020.587012. 10.3389/fmolb.2020.587012 PMC7726445 33324678

[13] Yezhelyev MV, Gao X, Xing Y, Al-Hajj A, Nie S, O’Regan RM (2006). Emerging use of nanoparticles in diagnosis and treatment of breast cancer. Lancet Oncol.

[14] Wang B, Wu W, Lu H, Wang Z, Xin H (2019). Enhanced anti-tumor of Pep-1 modified superparamagnetic iron oxide/PTX loaded polymer nanoparticles. Front Pharmacol.

[15] Gao N, Nie J, Wang H, Xing C, Mei L, Xiong W, Zeng X, Peng Z (2018). A versatile platform based on black phosphorus nanosheets with enhanced stability for cancer synergistic therapy. J Biomed Nanotechnol.

[16] Wang S-B, Ma Y-Y, Chen X-Y, Zhao Y-Y, Mou X-Z (2019). Ceramide-graphene oxide nanoparticles enhance cytotoxicity and decrease HCC xenograft development: a novel approach for targeted cancer therapy. Front Pharmacol.

[17] Singh P, Kim YJ, Singh H, Wang C, Hwang KH, Farh ME-A, Yang DC (2015). Biosynthesis, characterization, and antimicrobial applications of silver nanoparticles. Int J Nanomed.

[18] Pillai AM, Sivasankarapillai VS, Rahdar A, Joseph J, Sadeghfar F, Anuf AR, Rajesh K, Kyzas GZ (2020). Green synthesis and characterization of zinc oxide nanoparticles with antibacterial and antifungal activity. J Mol Struct.

[19] Murali M, Thampy A, Anandan S, Aiyaz M, Shilpa N, Singh SB, Gowtham HG, Ramesh AM, Rahdar A, Kyzas GZ (2023). Competent antioxidant and antiglycation properties of zinc oxide nanoparticles (ZnO-NPs) phyto-fabricated from aqueous leaf extract of Boerhaavia Erecta L.. Environ Sci Pollut Res.

[20] Pagar K, Chavan K, Kasav S, Basnet P, Rahdar A, Kataria N, Oza R, Abhale Y, Ravindran B, Pardeshi O (2023). Bio-inspired synthesis of CdO nanoparticles using Citrus Limetta peel extract and their diverse biomedical applications. J Drug Deliv Sci Technol..

[21] Dabhane H, Ghotekar S, Zate M, Lin K-YA, Rahdar A, Ravindran B, Bahiram D, Ingale C, Khairnar B, Sali D (2023). A novel approach toward the bio-inspired synthesis of CuO nanoparticles for phenol degradation and antimicrobial applications. Biomass Convers Biorefinery.

[22] Shava B, Ayodeji FD, Rahdar A, Iqbal HMN, Bilal M (2022). Magnetic nanoparticles-based systems for multifaceted biomedical applications. J Drug Deliv Sci Technol..

[23] Nowack B, Krug HF, Height M (2011). 120 Years of nanosilver history: implications for policy makers. Environ Sci Technol.

[24] Vaghari H, Jafarizadeh-Malmiri H, Mohammadlou M, Berenjian A, Anarjan N, Jafari N, Nasiri S (2016). Application of magnetic nanoparticles in smart enzyme immobilization. Biotechnol Lett.

[25] Orlowski P, Zmigrodzka M, Tomaszewska E, Ranoszek-Soliwoda K, Czupryn M, Antos-Bielska M, Szemraj J, Celichowski G, Grobelny J, Krzyzowska M (2018). Tannic acid-modified silver nanoparticles for wound healing: the importance of size. Int J Nanomed.

[26] Guilger-Casagrande M, Germano-Costa T, Bilesky-José N, Pasquoto-Stigliani T, Carvalho L, Fraceto LF, de Lima R (2021). Influence of the capping of biogenic silver nanoparticles on their toxicity and mechanism of action towards sclerotinia sclerotiorum. J Nanobiotechnol.

[27] Halkai KR, Mudda JA, Shivanna V, Rathod V, Halkai R (2018). Antibacterial efficacy of biosynthesized silver nanoparticles against Enterococcus Faecalis biofilm: an in vitro study. Contemp Clin Dent.

[28] Greenfeld JI, Sampath L, Popilskis SJ, Brunnert SR, Stylianos S, Modak S (1995). Decreased bacterial adherence and biofilm formation on chlorhexidine and silver sulfadiazine-impregnated central venous catheters implanted in swine. Crit Care Med.

[29] Barapatre A, Aadil KR, Jha H (2016). Synergistic antibacterial and antibiofilm activity of silver nanoparticles biosynthesized by lignin-degrading fungus. Bioresour Bioprocess.

[30] Kalishwaralal K, BarathManiKanth S, Pandian SRK, Deepak V, Gurunathan S (2010). Silver nanoparticles impede the biofilm formation by Pseudomonas Aeruginosa and Staphylococcus Epidermidis. Colloids Surf B Biointerfaces.

[31] Mohanty S, Mishra S, Jena P, Jacob B, Sarkar B, Sonawane A (2012). An investigation on the antibacterial, cytotoxic, and antibiofilm efficacy of starch-stabilized silver nanoparticles. Nanomed Nanotechnol Biol Med.

[32] Zhang W, Qiao X, Chen J (2006). Synthesis and characterization of silver nanoparticles in AOT microemulsion system. Chem Phys.

[33] Gholami A, Rasoul-amini S, Ebrahiminezhad A, Seradj SH, Ghasemi Y (2015). Lipoamino acid coated superparamagnetic iron oxide nanoparticles concentration and time dependently enhanced growth of human hepatocarcinoma cell line (Hep-G2). J Nanomater..

[34] Choi Y, Ryu GH, Min SH, Lee BR, Song MH, Lee Z, Kim B-S (2014). Interface-controlled synthesis of heterodimeric silver-carbon nanoparticles derived from polysaccharides. ACS Nano.

[35] Rizzello L, Pompa PP (2014). Nanosilver-based antibacterial drugs and devices: mechanisms, methodological drawbacks, and guidelines. Chem Soc Rev.

[36] Ebrahiminezhad A, Bagheri M, Taghizadeh S-M, Berenjian A, Ghasemi Y (2016). Biomimetic synthesis of silver nanoparticles using microalgal secretory carbohydrates as a novel anticancer and antimicrobial. Adv Nat Sci Nanosci Nanotechnol..

[37] Wang J, Li S, Han Y, Guan J, Chung S, Wang C, Li D (2018). Poly(ethylene glycol)–polylactide micelles for cancer therapy. Front Pharmacol..

[38] Calzoni E, Cesaretti A, Polchi A, Di Michele A, Tancini B, Emiliani C (2019). Biocompatible polymer nanoparticles for drug delivery applications in cancer and neurodegenerative disorder therapies. J Funct Biomater.

[39] Gouthami K, Lakshminarayana L, Faniband B, Veeraraghavan V, Bilal M, Bhargava RN, Ferreira LFR, Rahdar A, Kakkameli S, Mulla SI, Ali N, Bilal M, Khan A, Nguyen TA, Gupta RK (2023). 1—Introduction to polymeric nanomaterials. Smart polymer nanocomposites; micro and nano technologies.

[40] Kumar A, Sharipov M, Turaev A, Azizov S, Azizov I, Makhado E, Rahdar A, Kumar D, Pandey S (2022). Polymer-based hybrid nanoarchitectures for cancer therapy applications. Polymers.

[41] Rajput IB, Tareen FK, Khan AU, Ahmed N, Khan MFA, Shah KU, Rahdar A, Díez-Pascual AM (2023). Fabrication and in vitro evaluation of chitosan-gelatin based aceclofenac loaded scaffold. Int J Biol Macromol.

[42] Xu M, Liu J, Xu X, Liu S, Peterka F, Ren Y, Zhu X (2018). Synthesis and comparative biological properties of Ag-PEG nanoparticles with tunable morphologies from janus to multi-core shell structure. Materials.

[43] Wang F, Bao X, Fang A, Li H, Zhou Y, Liu Y, Jiang C, Wu J, Song X (2018). Nanoliposome-encapsulated brinzolamide-hydropropyl-β-cyclodextrin inclusion complex: a potential therapeutic ocular drug-delivery system. Front Pharmacol.

[44] Wang F, Xiao W, Elbahnasawy MA, Bao X, Zheng Q, Gong L, Zhou Y, Yang S, Fang A, Farag MMS (2018). Optimization of the linker length of mannose-cholesterol conjugates for enhanced MRNA delivery to dendritic cells by liposomes. Front Pharmacol.

[45] Yetisgin AA, Cetinel S, Zuvin M, Kosar A, Kutlu O (2020). Therapeutic nanoparticles and their targeted delivery applications. Molecules.

[46] Allen TM, Cullis PR (2013). Liposomal drug delivery systems: from concept to clinical applications. Adv Drug Deliv Rev.

[47] Daraee H, Etemadi A, Kouhi M, Alimirzalu S, Akbarzadeh A (2016). Application of liposomes in medicine and drug delivery. Artif Cells Nanomed Biotechnol.

[48] Buse J, El-Aneed A (2010). Properties, engineering and applications of lipid-based nanoparticle drug-delivery systems: current research and advances. Nanomed.

[49] Zhang H, Wang G, Yang H (2011). Drug delivery systems for differential release in combination therapy. Expert Opin Drug Deliv.

[50] Hsu H-J, Bugno J, Lee S-R, Hong S (2017). Dendrimer-based nanocarriers: a versatile platform for drug delivery. Wiley Interdiscip Rev Nanomed Nanobiotechnol..

[51] Palmerston Mendes L, Pan J, Torchilin VP (2017). Dendrimers as nanocarriers for nucleic acid and drug delivery in cancer therapy. Mol J Synth Chem Nat Prod Chem..

[52] Patel H, Patel P (2013). Dendrimer applications—a review. Int J Pharm Bio Sci.

[53] Maciejewski M (1982). Concepts of trapping topologically by shell molecules. J Macromol Sci.

[54] Bannas P, Hambach J, Koch-Nolte F (2017). Nanobodies and nanobody-based human heavy chain antibodies as antitumor therapeutics. Front Immunol.

[55] Hassanzadeh-Ghassabeh G, Devoogdt N, De Pauw P, Vincke C, Muyldermans S (2013). Nanobodies and their potential applications. Nanomedicine.

[56] Bao G, Tang M, Zhao J, Zhu X (2021). Nanobody: a promising toolkit for molecular imaging and disease therapy. EJNMMI Res.

[57] Nayak D, Kumari M, Rajachandar S, Ashe S, Thathapudi NC, Nayak B (2016). Biofilm impeding AgNPs target skin carcinoma by inducing mitochondrial membrane depolarization mediated through ROS production. ACS Appl Mater Interfaces.

[58] Ghosh S, Patil S, Ahire M, Kitture R, Kale S, Pardesi K, Cameotra SS, Bellare J, Dhavale DD, Jabgunde A (2012). Synthesis of silver nanoparticles using dioscorea bulbifera tuber extract and evaluation of its synergistic potential in combination with antimicrobial agents. Int J Nanomed.

[59] Hindi KM, Ditto AJ, Panzner MJ, Medvetz DA, Han DS, Hovis CE, Hilliard JK, Taylor JB, Yun YH, Cannon CL (2009). The antimicrobial efficacy of sustained release silver-carbene complex-loaded l-tyrosine polyphosphate nanoparticles: characterization, in vitro and in vivo studies. Biomaterials.

[60] Jena P, Mohanty S, Mallick R, Jacob B, Sonawane A (2012). Toxicity and antibacterial assessment of chitosancoated silver nanoparticles on human pathogens and macrophage cells. Int J Nanomed.

[61] Dakal TC, Kumar A, Majumdar RS, Yadav V (2016). Mechanistic basis of antimicrobial actions of silver nanoparticles. Front Microbiol..

[62] Gupta A, Maynes M, Silver S (1998). Effects of halides on plasmid-mediated silver resistance in Escherichia coli. Appl Environ Microbiol.

[63] Kim T, Braun GB, She Z, Hussain S, Ruoslahti E, Sailor MJ (2016). Composite porous silicon-silver nanoparticles as theranostic antibacterial agents. ACS Appl Mater Interfaces.

[64] Hoseinnejad M, Jafari SM, Katouzian I (2018). Inorganic and metal nanoparticles and their antimicrobial activity in food packaging applications. Crit Rev Microbiol.

[65] Feng QL, Wu J, Chen GQ, Cui FZ, Kim TN, Kim JO (2000). A mechanistic study of the antibacterial effect of silver ions on Escherichia coli and Staphylococcus aureus. J Biomed Mater Res.

[66] Betts AJ, Dowling DP, McConnell ML, Pope C (2005). The influence of platinum on the performance of silver-platinum anti-bacterial coatings. Mater Des.

[67] Mohamed Hamouda I (2012). Current perspectives of nanoparticles in medical and dental biomaterials. J Biomed Res.

[68] Rai M, Yadav A, Gade A (2009). Silver nanoparticles as a new generation of antimicrobials. Biotechnol Adv.

[69] Gunasekaran T, Nigusse T, Dhanaraju MD (2012). Silver nanoparticles as real topical bullets for wound healing. J Am Coll Clin Wound Spec.

[70] Wang L, Hu C, Shao L (2017). The antimicrobial activity of nanoparticles: present situation and prospects for the future. Int J Nanomed.

[71] Maeda H, Sawa T, Konno T (2001). Mechanism of tumor-targeted delivery of macromolecular drugs, including the EPR effect in solid tumor and clinical overview of the prototype polymeric drug SMANCS. J Control Release.

[72] Yan N, Xu J, Liu G, Ma C, Bao L, Cong Y, Wang Z, Zhao Y, Xu W, Chen C (2022). Penetrating macrophage-based nanoformulation for periodontitis treatment. ACS Nano.

[73] Alqahtani F, Aleanizy F, Tahir EE, Alhabib H, Alsaif R, Shazly G, AlQahtani H, Alsarra I, Mahdavi J (2020). Antibacterial activity of chitosan nanoparticles against pathogenic N. Gonorrhoea. Int J Nanomed.

[74] Ibrahim A, Moodley D, Uche C, Maboza E, Olivier A, Petrik L (2021). Antimicrobial and cytotoxic activity of electrosprayed chitosan nanoparticles against endodontic pathogens and Balb/c 3T3 fibroblast cells. Sci Rep.

[75] Oei JD, Zhao WW, Chu L, DeSilva MN, Ghimire A, Rawls HR, Whang K (2012). Antimicrobial acrylic materials with in situ generated silver nanoparticles. J Biomed Mater Res B Appl Biomater..

[76] Kim K-J, Sung WS, Suh BK, Moon S-K, Choi J-S, Kim JG, Lee DG (2009). Antifungal activity and mode of action of silver nano-particles on Candida albicans. Biometals.

[77] Nadworny PL, Wang J, Tredget EE, Burrell RE (2010). Anti-inflammatory activity of nanocrystalline silver-derived solutions in porcine contact dermatitis. J Inflamm.

[78] Lara HH, Ayala-Nuñez NV, Ixtepan-Turrent L, Rodriguez-Padilla C (2010). Mode of antiviral action of silver nanoparticles against HIV-1. J Nanobiotechnol.

[79] Freire PLL, Albuquerque AJR, Farias IAP, da Silva TG, Aguiar JS, Galembeck A, Flores MAP, Sampaio FC, Stamford TCM, Rosenblatt A (2016). Antimicrobial and cytotoxicity evaluation of colloidal chitosan – silver nanoparticles – fluoride nanocomposites. Int J Biol Macromol.

[80] Haitao Y, Yifan C, Mingchao S, Shuaijuan H (2022). A novel polymeric nanohybrid antimicrobial engineered by antimicrobial peptide MccJ25 and chitosan nanoparticles exerts strong antibacterial and anti-inflammatory activities. Front Immunol.

[81] Yu H, Ma Z, Meng S, Qiao S, Zeng X, Tong Z, Jeong KC (2021). A novel nanohybrid antimicrobial based on chitosan nanoparticles and antimicrobial peptide microcin J25 with low toxicity. Carbohydr Polym..

[82] Zhao Y, Sun X, Zhang G, Trewyn BG, Slowing II, Lin VS-Y (2011). Interaction of mesoporous silica nanoparticles with human red blood cell membranes: size and surface effects. ACS Nano.

[83] Lunov O, Syrovets T, Loos C, Beil J, Delacher M, Tron K, Nienhaus GU, Musyanovych A, Mailänder V, Landfester K (2011). Differential uptake of functionalized polystyrene nanoparticles by human macrophages and a monocytic cell line. ACS Nano.

[84] Maurer LL, Yang X, Schindler AJ, Taggart RK, Jiang C, Hsu-Kim H, Sherwood DR, Meyer JN (2016). Intracellular trafficking pathways in silver nanoparticle uptake and toxicity in Caenorhabditis Elegans. Nanotoxicology.

[85] Oh E, Delehanty JB, Sapsford KE, Susumu K, Goswami R, Blanco-Canosa JB, Dawson PE, Granek J, Shoff M, Zhang Q (2011). Cellular uptake and fate of PEGylated gold nanoparticles is dependent on both cell-penetration peptides and particle size. ACS Nano.

[86] Wang T, Zheng Y, Shi Y, Zhao L (2019). PH-responsive calcium alginate hydrogel laden with protamine nanoparticles and hyaluronan oligosaccharide promotes diabetic wound healing by enhancing angiogenesis and antibacterial activity. Drug Deliv Transl Res.

[87] Mihai MM, Dima MB, Dima B, Holban AM (2019). Nanomaterials for wound healing and infection control. Materials.

[88] Hamdan S, Pastar I, Drakulich S, Dikici E, Tomic-Canic M, Deo S, Daunert S (2017). Nanotechnology-driven therapeutic interventions in wound healing: potential uses and applications. ACS Cent Sci.

[89] Jaiswal M, Koul V, Dinda AKr (2016). In vitro and in vivo investigational studies of a nanocomposite-hydrogel-based dressing with a silver-coated chitosan wafer for full-thickness skin wounds. J Appl Polym Sci..

[90] Yan N, Hu B, Xu J, Cai R, Liu Z, Fu D, Huo B, Liu Z, Zhao Y, Chen C (2022). Stem cell Janus patch for periodontal regeneration. Nano Today.

[91] Xue J, Wu T, Dai Y, Xia Y (2019). Electrospinning and electrospun nanofibers: methods, materials, and applications. Chem Rev.

[92] Pal P, Dadhich P, Srivas PK, Das B, Maulik D, Dhara S (2017). Bilayered nanofibrous 3D hierarchy as skin rudiment by emulsion electrospinning for burn wound management. Biomater Sci.

[93] Tan G, Wang L, Pan W, Chen K (2022). Polysaccharide electrospun nanofibers for wound healing applications. Int J Nanomed.

[94] Pilehvar-Soltanahmadi Y, Akbarzadeh A, Moazzez-Lalaklo N, Zarghami N (2016). An update on clinical applications of electrospun nanofibers for skin bioengineering. Artif Cells Nanomed Biotechnol.

[95] Wu T, Xue J, Li H, Zhu C, Mo X, Xia Y (2018). General method for generating circular gradients of active proteins on nanofiber scaffolds sought for wound closure and related applications. ACS Appl Mater Interfaces.

[96] Zhou H, Lee J (2011). Nanoscale hydroxyapatite particles for bone tissue engineering. Acta Biomater.

[97] Wepener I, Richter W, van Papendorp D, Joubert AM (2012). In vitro osteoclast-like and osteoblast cells’ response to electrospun calcium phosphate biphasic candidate scaffolds for bone tissue engineering. J Mater Sci Mater Med.

[98] Song W, Markel DC, Wang S, Shi T, Mao G, Ren W (2012). Electrospun polyvinyl alcohol–collagen–hydroxyapatite nanofibers: a biomimetic extracellular matrix for osteoblastic cells. Nanotechnology.

[99] Baker BM, Nathan AS, Gee AO, Mauck RL (2010). The influence of an aligned nanofibrous topography on human mesenchymal stem cell fibrochondrogenesis. Biomaterials.

[100] Shafiee A, Soleimani M, Chamheidari GA, Seyedjafari E, Dodel M, Atashi A, Gheisari Y (2011). Electrospun nanofiber-based regeneration of cartilage enhanced by mesenchymal stem cells. J Biomed Mater Res A.

[101] Planka L, Srnec R, Rauser P, Stary D, Filova E, Jancar J, Juhasova J, Kren L, Necas A, Gal P (2012). Nanotechnology and mesenchymal stem cells with chondrocytes in prevention of partial growth plate arrest in pigs. Biomed Pap.

[102] You C, Li Q, Wang X, Wu P, Ho JK, Jin R, Zhang L, Shao H, Han C (2017). Silver nanoparticle loaded collagen/chitosan scaffolds promote wound healing via regulating fibroblast migration and macrophage activation. Sci Rep.

[103] Wright JB, Lam K, Buret AG, Olson ME, Burrell RE (2002). Early healing events in a porcine model of contaminated wounds: effects of nanocrystalline silver on matrix metalloproteinases, cell apoptosis, and healing. Wound Repair Regen..

[104] Gear AJ, Hellewell TB, Wright HR, Mazzarese PM, Arnold PB, Rodeheaver GT, Edlich RF (1997). A new silver sulfadiazine water soluble gel. Burns J Int Soc Burn Inj.

[105] Bowler PG, Welsby S, Towers V, Booth R, Hogarth A, Rowlands V, Joseph A, Jones SA (2012). Multidrug-resistant organisms, wounds and topical antimicrobial protection. Int Wound J.

[106] Liu X, Gan H, Hu C, Sun W, Zhu X, Meng Z, Gu R, Wu Z, Dou G (2018). Silver sulfadiazine nanosuspension-loaded thermosensitive hydrogel as a topical antibacterial agent. Int J Nanomed.

[107] Gao L, Zhou Y, Peng J, Xu C, Xu Q, Xing M, Chang J (2019). A novel dual-adhesive and bioactive hydrogel activated by bioglass for wound healing. NPG Asia Mater.

[108] Shi G, Chen W, Zhang Y, Dai X, Zhang X, Wu Z (2019). An antifouling hydrogel containing silver nanoparticles for modulating the therapeutic immune response in chronic wound healing. Langmuir ACS J Surf Colloids.

[109] Ballottin D, Fulaz S, Cabrini F, Tsukamoto J, Durán N, Alves OL, Tasic L (2017). Antimicrobial textiles: biogenic silver nanoparticles against Candida and Xanthomonas. Mater Sci Eng C Mater Biol Appl..

[110] Su C-H, Kumar GV, Adhikary S, Velusamy P, Pandian K, Anbu P (2017). Preparation of cotton fabric using sodium alginate-coated nanoparticles to protect against nosocomial pathogens. Biochem Eng J.

[111] Paladini F, Picca RA, Sportelli MC, Cioffi N, Sannino A, Pollini M (2015). Surface chemical and biological characterization of flax fabrics modified with silver nanoparticles for biomedical applications. Mater Sci Eng C Mater Biol Appl..

[112] Hua S, Wu SY (2013). The use of lipid-based nanocarriers for targeted pain therapies. Front Pharmacol.

[113] Ding B-S, Dziubla T, Shuvaev VV, Muro S, Muzykantov VR (2006). Advanced drug delivery systems that target the vascular endothelium. Mol Interv.

[114] Xu J, Zhang Y, Xu J, Liu G, Di C, Zhao X, Li X, Li Y, Pang N, Yang C (2020). Engineered nanoplatelets for targeted delivery of plasminogen activators to reverse thrombus in multiple mouse thrombosis models. Adv Mater.

[115] Valencia-Lazcano AA, Hassan D, Pourmadadi M, Shamsabadipour A, Behzadmehr R, Rahdar A, Medina DI, Díez-Pascual AM (2023). 5-Fluorouracil nano-delivery systems as a cutting-edge for cancer therapy. Eur J Med Chem..

[116] Pourmadadi M, Eshaghi MM, Rahmani E, Ajalli N, Bakhshi S, Mirkhaef H, Lasemi MV, Rahdar A, Behzadmehr R, Díez-Pascual AM (2022). Cisplatin-loaded nanoformulations for cancer therapy: a comprehensive review. J Drug Deliv Sci Technol..

[117] Rommasi F, Esfandiari N (2021). Liposomal nanomedicine: applications for drug delivery in cancer therapy. Nanoscale Res Lett.

[118] Olusanya TOB, Haj Ahmad RR, Ibegbu DM, Smith JR, Elkordy AA (2018). Liposomal drug delivery systems and anticancer drugs. Mol J Synth Chem Nat Prod Chem..

[119] Balzus B, Sahle FF, Hönzke S, Gerecke C, Schumacher F, Hedtrich S, Kleuser B, Bodmeier R (2017). Formulation and ex vivo evaluation of polymeric nanoparticles for controlled delivery of corticosteroids to the skin and the corneal epithelium. Eur J Pharm Biopharm.

[120] Braghirolli DI, Steffens D, Pranke P (2014). Electrospinning for regenerative medicine: a review of the main topics. Drug Discov Today.

[121] Sercombe L, Veerati T, Moheimani F, Wu SY, Sood AK, Hua S (2015). Advances and challenges of liposome assisted drug delivery. Front Pharmacol.

[122] Schwendener RA (2014). Liposomes as vaccine delivery systems: a review of the recent advances. Ther Adv Vaccines.

[123] Benyettou F, Rezgui R, Ravaux F, Jaber T, Blumer K, Jouiad M, Motte L, Olsen J-C, Platas-Iglesias C, Magzoub M (2015). Synthesis of silver nanoparticles for the dual delivery of doxorubicin and alendronate to cancer cells. J Mater Chem B.

[124] Brown PK, Qureshi AT, Moll AN, Hayes DJ, Monroe WT (2013). Silver nanoscale antisense drug delivery system for photoactivated gene silencing. ACS Nano.

[125] Naz M, Nasiri N, Ikram M, Nafees M, Qureshi MZ, Ali S, Tricoli A (2017). Eco-friendly biosynthesis, anticancer drug loading and cytotoxic effect of capped Ag-nanoparticles against breast cancer. Appl Nanosci.

[126] Park W, Na K (2015). Advances in the synthesis and application of nanoparticles for drug delivery. Wiley Interdiscip Rev Nanomed Nanobiotechnol.

[127] Khalid S, Hanif R (2017). Green biosynthesis of silver nanoparticles conjugated to gefitinib as delivery vehicle. Int J Adv Sci Eng Technol.

[128] Xu J, Zhang Y, Xu J, Wang M, Liu G, Wang J, Zhao X, Qi Y, Shi J, Cheng K (2019). Reversing tumor stemness via orally targeted nanoparticles achieves efficient colon cancer treatment. Biomaterials.

[129] Afsharzadeh M, Hashemi M, Babaei M, Abnous K, Ramezani M (2020). PEG-PLA nanoparticles decorated with small-molecule PSMA ligand for targeted delivery of galbanic acid and docetaxel to prostate cancer cells. J Cell Physiol.

[130] Pamujula S, Hazari S, Bolden G, Graves RA, Chinta DD, Dash S, Kishore V, Mandal TK (2012). Cellular delivery of PEGylated PLGA nanoparticles. J Pharm Pharmacol.

[131] Kościk I, Jankowski D, Jagusiak A. Carbon nanomaterials for theranostic use. C. 2022; 8:3. 10.3390/c8010003.

[132] Kearns O, Camisasca A, Giordani S (2021). Hyaluronic acid-conjugated carbon nanomaterials for enhanced tumour targeting ability. Molecules.

[133] Giusto E, Žárská L, Beirne DF, Rossi A, Bassi G, Ruffini A, Montesi M, Montagner D, Ranc V, Panseri S (2022). Graphene oxide nanoplatforms to enhance cisplatin-based drug delivery in anticancer therapy. Nanomaterials.

[134] Oberoi HS, Nukolova NV, Kabanov AV, Bronich TK (2013). Nanocarriers for delivery of platinum anticancer drugs. Adv Drug Deliv Rev.

[135] Qian Q, Zhu L, Zhu X, Sun M, Yan D (2019). Drug-polymer hybrid macromolecular engineering: degradable PEG integrated by Platinum(IV) for cancer therapy. Matter.

[136] Xiao X, Wang T, Li L, Zhu Z, Zhang W, Cui G, Li W (2019). Co-delivery of cisplatin(IV) and capecitabine as an effective and non-toxic cancer treatment. Front Pharmacol..

[137] Dong Z, Kang Y, Yuan Q, Luo M, Gu Z (2018). H2O2-responsive nanoparticle based on the supramolecular self-assemble of cyclodextrin. Front Pharmacol..

[138] Xiong Q, Cui M, Yu G, Wang J, Song T (2018). Facile fabrication of reduction-responsive supramolecular nanoassemblies for co-delivery of doxorubicin and sorafenib toward hepatoma cells. Front Pharmacol..

[139] Cuong N-V, Jiang J-L, Li Y-L, Chen J-R, Jwo S-C, Hsieh M-F (2010). Doxorubicin-loaded PEG-PCL-PEG micelle using xenograft model of nude mice: effect of multiple administration of micelle on the suppression of human breast cancer. Cancers.

[140] Behl A, Solanki S, Paswan SK, Datta TK, Saini AK, Saini RV, Parmar VS, Thakur VK, Malhotra S, Chhillar AK (2022). Biodegradable PEG-PCL nanoparticles for co-delivery of MUC1 inhibitor and doxorubicin for the confinement of triple-negative breast cancer. J Polym Environ.

[141] Ahmad Shariff SH, Wan Abdul Khodir WK, Abd Hamid S, Haris MS, Ismail MW (2022). Poly(Caprolactone)-b-Poly(Ethylene Glycol)-based polymeric micelles as drug carriers for efficient breast cancer therapy: a systematic review. Polymers.

[142] Xiang Z, Guan X, Ma Z, Shi Q, Panteleev M, Ataullakhanov FI (2022). Bioactive engineered scaffolds based on PCL-PEG-PCL and tumor cell-derived exosomes to minimize the foreign body reaction. Biomater Biosyst..

[143] Niu K, Yao Y, Xiu M, Guo C, Ge Y, Wang J (2018). Controlled drug delivery by polylactide Stereocomplex Micelle for cervical cancer chemotherapy. Front Pharmacol..

[144] Zhu Y-H, Ye N, Tang X-F, Khan MI, Liu H-L, Shi N, Hang L-F (2019). Synergistic effect of retinoic acid polymeric micelles and prodrug for the pharmacodynamic evaluation of tumor suppression. Front Pharmacol.

[145] Kong N, Deng M, Sun X-N, Chen Y-D, Sui X-B (2018). Polydopamine-functionalized CA-(PCL-Ran-PLA) nanoparticles for target delivery of docetaxel and chemo-photothermal therapy of breast cancer. Front Pharmacol..

[146] Chen X, Zhao L, Kang Y, He Z, Xiong F, Ling X, Wu J (2018). Significant suppression of non-small-cell lung cancer by hydrophobic poly(ester amide) nanoparticles with high docetaxel loading. Front Pharmacol..

[147] Nan W, Ding L, Chen H, Khan FU, Yu L, Sui X, Shi X (2018). Topical use of quercetin-loaded chitosan nanoparticles against ultraviolet B radiation. Front Pharmacol..

[148] Feng C, Zhu D, Chen L, Lu Y, Liu J, Kim NY, Liang S, Zhang X, Lin Y, Ma Y (2019). Targeted delivery of chlorin E6 via redox sensitive diselenide-containing micelles for improved photodynamic therapy in cluster of differentiation 44-overexpressing breast cancer. Front Pharmacol.

[149] Wu J, Yuan J, Ye B, Wu Y, Xu Z, Chen J, Chen J (2018). Dual-responsive core crosslinking glycopolymer-drug conjugates nanoparticles for precise hepatocarcinoma therapy. Front Pharmacol..

[150] Faid AH, Shouman SA, Badr YA, Sharaky M (2022). Enhanced photothermal heating and combination therapy of gold nanoparticles on a breast cell model. BMC Chem.

[151] Huo S, Ma H, Huang K, Liu J, Wei T, Jin S, Zhang J, He S, Liang X-J (2013). Superior penetration and retention behavior of 50 Nm gold nanoparticles in tumors. Cancer Res.

[152] Roudsari MH, Saeidi N, Kabiri N, Ahmadi A, Tabrizi MM, Shahmabadi HE, Khiyavi AA, Reghbati B (2016). Investigation of characteristics and behavior of loaded carboplatin on the, liposomes nanoparticles, on the lung and ovarian cancer: an in-vitro evaluation. Asian Pac J Cancer Biol.

[153] Lomis N, Westfall S, Farahdel L, Malhotra M, Shum-Tim D, Prakash S (2016). Human serum albumin nanoparticles for use in cancer drug delivery: process optimization and in vitro characterization. Nanomaterials.

[154] Zhao L, Zhao W, Liu Y, Chen X, Wang Y (2017). Nano-hydroxyapatite-derived drug and gene co-delivery system for anti-angiogenesis therapy of breast cancer. Med Sci Monit.

[155] Chiu HI, Samad NA, Fang L, Lim V (2021). Cytotoxicity of targeted PLGA nanoparticles: a systematic review. RSC Adv.

[156] Abdellatif AAH, Ali AT, Bouazzaoui A, Alsharidah M, Rugaie OA, Tolba NS (2022). Formulation of polymeric nanoparticles loaded sorafenib; evaluation of cytotoxicity, molecular evaluation, and gene expression studies in lung and breast cancer cell lines. Nanotechnol Rev.

[157] Vangara KK, Liu JL, Palakurthi S (2013). Hyaluronic acid-decorated PLGA-PEG nanoparticles for targeted delivery of SN-38 to ovarian cancer. Anticancer Res.

[158] Jin C, Wang S, Bai L. Preparation of paclitaxel-loaded nanoparticles targeting liver cancer stem cells and their effects on liver cancer Huh-7 and HepG2 cells. Cancer Res Clin. 2021;(6):99–103.

[159] Dey SK, Mandal B, Bhowmik M, Ghosh LK (2009). Development and in vitro evaluation of letrozole loaded biodegradable nanoparticles for breast cancer therapy. Braz J Pharm Sci.

[160] Zhang R, Ru Y, Gao Y, Li J, Mao S (2017). Layer-by-layer nanoparticles co-loading gemcitabine and platinum (IV) prodrugs for synergistic combination therapy of lung cancer. Drug Des Devel Therapy.

[161] Nokhodi F, Nekoei M, Goodarzi MT (2022). Hyaluronic acid-coated chitosan nanoparticles as targeted-carrier of tamoxifen against MCF7 and TMX-resistant MCF7 cells. J Mater Sci Mater Med.

[162] Shah HS, Joshi SA, Haider A, Kortz U, ur-Rehman N, Iqbal J (2015). Synthesis of chitosan-coated polyoxometalate nanoparticles against cancer and its metastasis. RSC Adv.

[163] Comparetti EJ, Lins PMP, Quitiba JVB, Zucolotto V (2020). Cancer cell membrane-derived nanoparticles improve the activity of gemcitabine and paclitaxel on pancreatic cancer cells and coordinate immunoregulatory properties on professional antigen-presenting cells. Mater Adv.

[164] Barenholz Y (2012). Doxil®—the first FDA-approved nano-drug: lessons learned. J Control Rel.

[165] O’Brien S, Schiller G, Lister J, Damon L, Goldberg S, Aulitzky W, Ben-Yehuda D, Stock W, Coutre S, Douer D (2013). High-dose vincristine sulfate liposome injection for advanced, relapsed, and refractory adult Philadelphia chromosome-negative acute lymphoblastic leukemia. J Clin Oncol.

[166] Silverman JA, Deitcher SR (2013). Marqibo® (Vincristine Sulfate Liposome Injection) improves the pharmacokinetics and pharmacodynamics of vincristine. Cancer Chemother Pharmacol.

[167] Cohen SM, Rockefeller N, Mukerji R, Durham D, Forrest ML, Cai S, Cohen MS, Shnayder Y (2013). Efficacy and toxicity of peritumoral delivery of nanoconjugated cisplatin in an in vivo murine model of head and neck squamous cell carcinoma. JAMA Otolaryngol Head Neck Surg..

[168] Stathopoulos GP, Boulikas T (2012). Lipoplatin formulation review article. J Drug Deliv.

[169] Boulikas T (2004). Low toxicity and anticancer activity of a novel liposomal cisplatin (lipoplatin) in mouse xenografts. Oncol Rep.

[170] Boulikas T (2009). Clinical overview on lipoplatin: a successful liposomal formulation of cisplatin. Expert Opin Investig Drugs.

[171] Farhat FS, Temraz S, Kattan J, Ibrahim K, Bitar N, Haddad N, Jalloul R, Hatoum HA, Nsouli G, Shamseddine AI (2011). A phase II study of lipoplatin (liposomal cisplatin)/vinorelbine combination in HER-2/Neu-negative metastatic breast cancer. Clin Breast Cancer.

[172] Panowski S, Bhakta S, Raab H, Polakis P, Junutula JR (2014). Site-specific antibody drug conjugates for cancer therapy. MAbs.

[173] Chen L, Wang L, Shion H, Yu C, Yu YQ, Zhu L, Li M, Chen W, Gao K (2016). In-depth structural characterization of Kadcyla® (ado-trastuzumab emtansine) and its biosimilar candidate. MAbs.

[174] Xu Z, Guo D, Jiang Z, Tong R, Jiang P, Bai L, Chen L, Zhu Y, Guo C, Shi J (2019). Novel HER2-targeting antibody-drug conjugates of trastuzumab beyond T-DM1 in breast cancer: Trastuzumab Deruxtecan(DS-8201a) and (Vic-)Trastuzumab Duocarmazine (SYD985). Eur J Med Chem..

[175] Gradishar WJ (2006). Albumin-bound paclitaxel: a next-generation taxane. Expert Opin Pharmacother.

[176] Jeyaraj M, Rajesh M, Arun R, MubarakAli D, Sathishkumar G, Sivanandhan G, Dev GK, Manickavasagam M, Premkumar K, Thajuddin N (2013). An investigation on the cytotoxicity and caspase-mediated apoptotic effect of biologically synthesized silver nanoparticles using podophyllum hexandrum on human cervical carcinoma cells. Colloids Surf B Biointerfaces.

[177] Kelly KL, Coronado E, Zhao LL, Schatz GC (2003). The optical properties of metal nanoparticles: the influence of size, shape, and dielectric environment. J Phys Chem B.

[178] Khan I, Saeed K, Khan I (2019). Nanoparticles: properties, applications and toxicities. Arab J Chem.

[179] Sharma V, Verma D, Okram GS (2020). Influence of surfactant, particle size and dispersion medium on surface plasmon resonance of silver nanoparticles. J Phys Condens Matter.

[180] Alexis F, Pridgen E, Molnar LK, Farokhzad OC (2008). Factors affecting the clearance and biodistribution of polymeric nanoparticles. Mol Pharm.

[181] Galić E, Ilić K, Hartl S, Tetyczka C, Kasemets K, Kurvet I, Milić M, Barbir R, Pem B, Erceg I (2020). Impact of surface functionalization on the toxicity and antimicrobial effects of selenium nanoparticles considering different routes of entry. Food Chem Toxicol..

[182] Piktel E, Niemirowicz K, Wątek M, Wollny T, Deptuła P, Bucki R (2016). Recent insights in nanotechnology-based drugs and formulations designed for effective anti-cancer therapy. J Nanobiotechnol.

[183] Peer D, Karp JM, Hong S, Farokhzad OC, Margalit R, Langer R (2007). Nanocarriers as an emerging platform for cancer therapy. Nat Nanotechnol.

[184] Panyam J, Labhasetwar V (2012). Biodegradable nanoparticles for drug and gene delivery to cells and tissue. Adv Drug Deliv Rev.

[185] He X, Ma J, Mercado AE, Xu W, Jabbari E (2008). Cytotoxicity of paclitaxel in biodegradable self-assembled core-shell poly(lactide-co-glycolide ethylene oxide fumarate) nanoparticles. Pharm Res.

[186] George BPA, Kumar N, Abrahamse H, Ray SS (2018). Apoptotic efficacy of multifaceted biosynthesized silver nanoparticles on human adenocarcinoma cells. Sci Rep.

[187] Ramar M, Manikandan B, Marimuthu PN, Raman T, Mahalingam A, Subramanian P, Karthick S, Munusamy A (2015). Synthesis of silver nanoparticles using solanum trilobatum fruits extract and its antibacterial, cytotoxic activity against human breast cancer cell line MCF 7. Spectrochim Acta A Mol Biomol Spectrosc.

[188] Venugopal K, Rather HA, Rajagopal K, Shanthi MP, Sheriff K, Illiyas M, Rather RA, Manikandan E, Uvarajan S, Bhaskar M (2017). Synthesis of silver nanoparticles (Ag NPs) for anticancer activities (MCF 7 breast and A549 lung cell lines) of the crude extract of Syzygium Aromaticum. J Photochem Photobiol B.

[189] Kikuchi M, Kuroki S, Kayama M, Sakaguchi S, Lee K-K, Yonehara S (2012). Protease activity of procaspase-8 is essential for cell survival by inhibiting both apoptotic and nonapoptotic cell death dependent on receptor-interacting protein kinase 1 (RIP1) and RIP3 *. J Biol Chem.

[190] Selvi BCG, Madhavan J, Santhanam A (2016). Cytotoxic effect of silver nanoparticles synthesized from padina tetrastromatica on breast cancer cell line. Adv Nat Sci Nanosci Nanotechnol.

[191] Bin-Jumah M, Al-Abdan M, Albasher G, Alarifi S (2020). Effects of green silver nanoparticles on apoptosis and oxidative stress in normal and cancerous human hepatic cells in vitro. Int J Nanomed..

[192] Arora S, Jain J, Rajwade JM, Paknikar KM (2008). Cellular responses induced by silver nanoparticles. In Vitro Stud Toxicol Lett.

[193] Ullah I, Khalil AT, Ali M, Iqbal J, Ali W, Alarifi S, Shinwari ZK (2020). Green-synthesized silver nanoparticles induced apoptotic cell death in MCF-7 breast cancer cells by generating reactive oxygen species and activating caspase 3 and 9 enzyme activities. Oxid Med Cell Longev..

[194] Wyllie AH (1980). Glucocorticoid-induced thymocyte apoptosis is associated with endogenous endonuclease activation. Nature.

[195] Zhang P, Meng J, Li Y, Yang C, Hou Y, Tang W, McHugh KJ, Jing L (2021). Nanotechnology-enhanced immunotherapy for metastatic cancer. Innovation.

[196] Goldberg MS (2019). Improving cancer immunotherapy through nanotechnology. Nat Rev Cancer.

[197] Khalil DN, Smith EL, Brentjens RJ, Wolchok JD (2016). The future of cancer treatment: immunomodulation, CARs and combination immunotherapy. Nat Rev Clin Oncol.

[198] Hickey JW, Vicente FP, Howard GP, Mao H-Q, Schneck JP (2017). Biologically inspired design of nanoparticle artificial antigen-presenting cells for immunomodulation. Nano Lett.

[199] Stephan MT, Stephan SB, Bak P, Chen J, Irvine DJ (2012). Synapse-directed delivery of immunomodulators using T-cell-conjugated nanoparticles. Biomaterials.

[200] Radovic-Moreno AF, Chernyak N, Mader CC, Nallagatla S, Kang RS, Hao L, Walker DA, Halo TL, Merkel TJ, Rische CH (2015). Immunomodulatory spherical nucleic acids. Proc Natl Acad Sci.

[201] Zheng Y, Tang L, Mabardi L, Kumari S, Irvine DJ (2017). Enhancing adoptive cell therapy of cancer through targeted delivery of small-molecule immunomodulators to internalizing or noninternalizing receptors. ACS Nano.

[202] Li J, Luo Y, Zeng Z, Cui D, Huang J, Xu C, Li L, Pu K, Zhang R (2022). Precision cancer sono-immunotherapy using deep-tissue activatable semiconducting polymer immunomodulatory nanoparticles. Nat Commun.

[203] Adams GP, Weiner LM (2005). Monoclonal antibody therapy of cancer. Nat Biotechnol.

[204] Kimiz-Gebologlu I, Gulce-Iz S, Biray-Avci C (2018). Monoclonal antibodies in cancer immunotherapy. Mol Biol Rep.

[205] Weiner LM, Dhodapkar MV, Ferrone S (2009). Monoclonal antibodies for cancer immunotherapy. Lancet.

[206] Pardoll DM (2012). The blockade of immune checkpoints in cancer immunotherapy. Nat Rev Cancer.

[207] Ribas A, Wolchok JD (2018). Cancer immunotherapy using checkpoint blockade. Science.

[208] Berraondo P, Sanmamed MF, Ochoa MC, Etxeberria I, Aznar MA, Pérez-Gracia JL, Rodríguez-Ruiz ME, Ponz-Sarvise M, Castañón E, Melero I (2019). Cytokines in clinical cancer immunotherapy. Br J Cancer.

[209] Lee S, Margolin K (2011). Cytokines in cancer immunotherapy. Cancers.

[210] Hemminki O, dos Santos JM, Hemminki A (2020). Oncolytic viruses for cancer immunotherapy. J Hematol Oncol.

[211] Liu S, Galat V, Galat Y, Lee YKA, Wainwright D, Wu J (2021). NK cell-based cancer immunotherapy: from basic biology to clinical development. J Hematol Oncol.

[212] Shin MH, Kim J, Lim SA, Kim J, Kim S-J, Lee K-M (2020). NK cell-based immunotherapies in cancer. Immune Netw..

[213] Cerwenka A, Lanier LL (2016). Natural killer cell memory in infection, inflammation and cancer. Nat Rev Immunol.

[214] Guillerey C, Huntington ND, Smyth MJ (2016). Targeting natural killer cells in cancer immunotherapy. Nat Immunol.

[215] Maude SL, Frey N, Shaw PA, Aplenc R, Barrett DM, Bunin NJ, Chew A, Gonzalez VE, Zheng Z, Lacey SF (2014). Chimeric antigen receptor T cells for sustained remissions in leukemia. N Engl J Med.

[216] Kalos M, Levine BL, Porter DL, Katz S, Grupp SA, Bagg A, June CH (2011). T cells with chimeric antigen receptors have potent antitumor effects and can establish memory in patients with advanced leukemia. Sci Transl Med..

[217] Markov OV, Mironova NL, Sennikov SV, Vlassov VV, Zenkova MA (2015). Prophylactic dendritic cell-based vaccines efficiently inhibit metastases in murine metastatic melanoma. PLoS ONE.

[218] Zhang Y, Lin S, Wang X-Y, Zhu G (2019). Nanovaccines for cancer immunotherapy. Wiley Interdiscip Rev Nanomed Nanobiotechnol..

[219] Miao L, Zhang Y, Huang L (2021). MRNA vaccine for cancer immunotherapy. Mol Cancer.

[220] Salem ML, Lawman MJP, Lawman PD (2014). The use of dendritic cells for peptide-based vaccination in cancer immunotherapy. Cancer vaccines: methods and protocols; methods in molecular biology.

[221] Palucka K, Banchereau J (2012). Cancer immunotherapy via dendritic cells. Nat Rev Cancer.

[222] Ahmed MS, Bae Y-S (2014). Dendritic cell-based therapeutic cancer vaccines: past, present and future. Clin Exp Vaccine Res.

[223] Palucka K, Ueno H, Fay J, Banchereau J (2011). Dendritic cells and immunity against cancer. J Intern Med.

[224] Upadhyay S, Sharma N, Gupta KB, Dhiman M (2018). Role of immune system in tumor progression and carcinogenesis. J Cell Biochem.

[225] Salemme V, Centonze G, Cavallo F, Defilippi P, Conti L. The crosstalk between tumor cells and the immune microenvironment in breast cancer: implications for immunotherapy. Front Oncol. 2021;11:610303. 10.3389/fonc.2021.610303. 10.3389/fonc.2021.610303 PMC7991834 33777750

[226] Gonzalez H, Hagerling C, Werb Z (2018). Roles of the immune system in cancer: from tumor initiation to metastatic progression. Genes Dev.

[227] Pinto A, Pocard M (2018). Photodynamic therapy and photothermal therapy for the treatment of peritoneal metastasis: a systematic review. Pleura Peritoneum.

[228] Li X, Lovell JF, Yoon J, Chen X (2020). Clinical development and potential of photothermal and photodynamic therapies for cancer. Nat Rev Clin Oncol.

[229] Kong C, Chen X (2022). Combined photodynamic and photothermal therapy and immunotherapy for cancer treatment: a review. Int J Nanomed.

[230] Guo S, Song Z, Ji D-K, Reina G, Fauny J-D, Nishina Y, Ménard-Moyon C, Bianco A (2022). Combined photothermal and photodynamic therapy for cancer treatment using a multifunctional graphene oxide. Pharmaceutics.

[231] Li R-T, Zhu Y-D, Li W-Y, Hou Y-K, Zou Y-M, Zhao Y-H, Zou Q, Zhang W-H, Chen J-X (2022). Synergistic photothermal-photodynamic-chemotherapy toward breast cancer based on a liposome-coated core-shell AuNS@NMOFs nanocomposite encapsulated with gambogic acid. J Nanobiotechnol.

[232] Liu P, Yang W, Shi L, Zhang H, Xu Y, Wang P, Zhang G, Chen WR, Zhang B, Wang X (2019). Concurrent photothermal therapy and photodynamic therapy for cutaneous squamous cell carcinoma by gold nanoclusters under a single NIR laser irradiation. J Mater Chem B.

[233] Shibu ES, Hamada M, Murase N, Biju V (2013). Nanomaterials formulations for photothermal and photodynamic therapy of cancer. J Photochem Photobiol C Photochem Rev.

[234] Dougherty TJ, Gomer CJ, Henderson BW, Jori G, Kessel D, Korbelik M, Moan J, Peng Q (1998). Photodynamic therapy. J Natl Cancer Inst.

[235] Li W, Yang J, Luo L, Jiang M, Qin B, Yin H, Zhu C, Yuan X, Zhang J, Luo Z (2019). Targeting photodynamic and photothermal therapy to the endoplasmic reticulum enhances immunogenic cancer cell death. Nat Commun.

[236] Chen Q, Xu L, Liang C, Wang C, Peng R, Liu Z (2016). Photothermal therapy with immune-adjuvant nanoparticles together with checkpoint blockade for effective cancer immunotherapy. Nat Commun.

[237] Santos LL, Oliveira J, Monteiro E, Santos J, Sarmento C (2018). Treatment of head and neck cancer with photodynamic therapy with redaporfin: a clinical case report. Case Rep Oncol.

[238] Chu KF, Dupuy DE (2014). Thermal ablation of tumours: biological mechanisms and advances in therapy. Nat Rev Cancer.

[239] Nyst HJ, Tan IB, Stewart FA, Balm AJM (2009). Is photodynamic therapy a good alternative to surgery and radiotherapy in the treatment of head and neck cancer?. Photodiagnosis Photodyn Ther.

[240] Allison RR, Sibata CH, Downie GH, Cuenca RE (2006). A clinical review of PDT for cutaneous malignancies. Photodiagn Photodyn Therapy.

[241] Zhao Y, Liu X, Liu X, Yu J, Bai X, Wu X, Guo X, Liu Z, Liu X (2022). Combination of phototherapy with immune checkpoint blockade: theory and practice in cancer. Front Immunol.

[242] Naylor MF, Chen WR, Teague TK, Perry LA, Nordquist RE (2006). In situ photoimmunotherapy: a tumour-directed treatment for melanoma. Br J Dermatol.

[243] Mroz P, Hashmi JT, Huang Y-Y, Lange N, Hamblin MR (2011). Stimulation of anti-tumor immunity by photodynamic therapy. Expert Rev Clin Immunol.

[244] Lucky SS, Soo KC, Zhang Y (2015). Nanoparticles in photodynamic therapy. Chem Rev.

[245] Yu J, Yin W, Zheng X, Tian G, Zhang X, Bao T, Dong X, Wang Z, Gu Z, Ma X (2015). Smart MoS_2_/Fe_3_O_4_ nanotheranostic for magnetically targeted photothermal therapy guided by magnetic resonance/photoacoustic imaging. Theranostics.

[246] Zhou Z, Sun Y, Shen J, Wei J, Yu C, Kong B, Liu W, Yang H, Yang S, Wang W (2014). Iron/iron oxide core/shell nanoparticles for magnetic targeting MRI and near-infrared photothermal therapy. Biomaterials.

[247] Bolze F, Jenni S, Sour A, Heitz V (2017). Molecular photosensitisers for two-photon photodynamic therapy. Chem Commun.

[248] Chen G, Qiu H, Prasad PN, Chen X (2014). Upconversion nanoparticles: design, nanochemistry, and applications in theranostics. Chem Rev.

[249] Hou X, Tao Y, Pang Y, Li X, Jiang G, Liu Y (2018). Nanoparticle-based photothermal and photodynamic immunotherapy for tumor treatment. Int J Cancer.

[250] Guo W, Chen Z, Chen J, Feng X, Yang Y, Huang H, Liang Y, Shen G, Liang Y, Peng C (2020). Biodegradable hollow mesoporous organosilica nanotheranostics (HMON) for multi-mode imaging and mild photo-therapeutic-induced mitochondrial damage on gastric cancer. J Nanobiotechnol.

[251] Zou J, Li L, Yang Z, Chen X (2021). Phototherapy meets immunotherapy: a win-win strategy to fight against cancer. Nanophotonics.

[252] Mew D, Wat CK, Towers GH, Levy JG (1950). Photoimmunotherapy: treatment of animal tumors with tumor-specific monoclonal antibody-hematoporphyrin conjugates. J Immunol Baltim Md.

[253] Cross D, Burmester JK (2006). Gene therapy for cancer treatment: past, present and future. Clin Med Res.

[254] Belete TM (2021). The current status of gene therapy for the treatment of cancer. Biol Targets Therapy.

[255] Weichselbaum RR, Kufe D (1997). Gene therapy of cancer. Lancet.

[256] Gaj T, Sirk SJ, Shui S, Liu J (2016). Genome-editing technologies: principles and applications. Cold Spring Harb Perspect Biol..

[257] Montaño-Samaniego M, Bravo-Estupiñan DM, Méndez-Guerrero O, Alarcón-Hernández E, Ibáñez-Hernández M (2020). Strategies for targeting gene therapy in cancer cells with tumor-specific promoters. Front Oncol.

[258] Gonçalves GAR, Paiva RMA (2017). Gene therapy: advances challenges and perspectives. Einstein.

[259] Roma-Rodrigues C, Rivas-García L, Baptista PV, Fernandes AR (2020). Gene therapy in cancer treatment: why go nano?. Pharmaceutics.

[260] Wang K, Kievit FM, Zhang M (2016). Nanoparticles for cancer gene therapy: recent advances, challenges, and strategies. Pharmacol Res.

[261] Roacho-Perez JA, Gallardo-Blanco HL, Sanchez-Dominguez M, Garcia-Casillas PE, Chapa-Gonzalez C, Sanchez-Dominguez CN (2018). Nanoparticles for death-induced gene therapy in cancer (review). Mol Med Rep.

[262] Lin G, Zhang H, Huang L (2015). Smart polymeric nanoparticles for cancer gene delivery. Mol Pharm.

[263] Mangraviti A, Tzeng SY, Kozielski KL, Wang Y, Jin Y, Gullotti D, Pedone M, Buaron N, Liu A, Wilson DR (2015). Polymeric nanoparticles for nonviral gene therapy extend brain tumor survival in vivo. ACS Nano.

[264] Saga K, Kaneda Y (2013). Virosome presents multimodel cancer therapy without viral replication. BioMed Res Int.

[265] Kaneda Y (2012). Virosome: a novel vector to enable multi-modal strategies for cancer therapy. Adv Drug Deliv Rev.

[266] Yamada T, Iwasaki Y, Tada H, Iwabuki H, Chuah MKL, VandenDriessche T, Fukuda H, Kondo A, Ueda M, Seno M (2003). Nanoparticles for the delivery of genes and drugs to human hepatocytes. Nat Biotechnol.

[267] López AG. Nanotechnology and autoimmunity. El Rosario University Press, 2013. 29087650

[268] Serra P, Santamaria P (2015). Nanoparticle-based autoimmune disease therapy. Clin Immunol Orlando Fla.

[269] He R, Li L, Zhang T, Ding X, Xing Y, Zhu S, Gu Z, Hu H (2023). Recent advances of nanotechnology application in autoimmune diseases—a bibliometric analysis. Nano Today.

[270] Rahimizadeh P, Rezaieyazdi Z, Behzadi F, Hajizade A, Lim SI (2021). Nanotechnology as a promising platform for rheumatoid arthritis management: diagnosis, treatment, and treatment monitoring. Int J Pharm.

[271] Fotooh Abadi L, Damiri F, Zehravi M, Joshi R, Pai R, Berrada M, Massoud EES, Rahman MH, Rojekar S, Cavalu S (2022). Novel nanotechnology-based approaches for targeting HIV reservoirs. Polymers.

[272] Cao S, Woodrow KA (2019). Nanotechnology approaches to eradicating HIV reservoirs. Eur J Pharm Biopharm..

[273] Lim H, Lee SH, Lee HT, Lee JU, Son JY, Shin W, Heo Y-S (2018). Structural biology of the TNFα antagonists used in the treatment of rheumatoid arthritis. Int J Mol Sci.

[274] Horton S, Walsh C, Emery P (2012). Certolizumab pegol for the treatment of rheumatoid arthritis. Expert Opin Biol Ther.

[275] Yudoh K, Karasawa R, Masuko K, Kato T (2009). Water-soluble fullerene (C60) inhibits the development of arthritis in the rat model of arthritis. Int J Nanomed.

[276] de Castro S, Camarasa M-J (2018). Polypharmacology in HIV inhibition: can a drug with simultaneous action against two relevant targets be an alternative to combination therapy?. Eur J Med Chem.

[277] Herskovitz J, Gendelman HE (2019). HIV and the macrophage: from cell reservoirs to drug delivery to viral eradication. J Neuroimmune Pharmacol..

[278] Dutta T, Garg M, Jain NK (2008). Targeting of efavirenz loaded tuftsin conjugated poly(propyleneimine) dendrimers to HIV infected macrophages in vitro. Eur J Pharm Sci.

[279] Prabhu S, Poulose EK (2012). Silver nanoparticles: mechanism of antimicrobial action, synthesis, medical applications, and toxicity effects. Int Nano Lett.

[280] Mahendiran B, Azeez NA, Muthusamy S, Krishnakumar GS, Barhoum A, Jeevanandam J, Danquah MK (2022). Chapter 9—polymer-based bionanomaterials for targeted drug delivery. Fundamentals of bionanomaterials; micro and nano technologies.

[281] Wilczewska AZ, Niemirowicz K, Markiewicz KH, Car H (2012). Nanoparticles as drug delivery systems. Pharmacol Rep.

[282] Valodkar M, Rathore PS, Jadeja RN, Thounaojam M, Devkar RV, Thakore S (2012). Cytotoxicity evaluation and antimicrobial studies of starch capped water soluble copper nanoparticles. J Hazard Mater.

[283] Pereira RF, Barrias CC, Granja PL, Bartolo PJ (2013). Advanced biofabrication strategies for skin regeneration and repair. Nanomed.

[284] Boateng JS, Matthews KH, Stevens HNE, Eccleston GM (2008). Wound healing dressings and drug delivery systems: a review. J Pharm Sci.

[285] Jurczak F, Dugré T, Johnstone A, Offori T, Vujovic Z, Hollander D (2007). Randomised clinical trial of hydrofiber dressing with silver versus povidone-iodine gauze in the management of open surgical and traumatic wounds. Int Wound J.

[286] Nayak PS, Pradhan S, Arakha M, Kumar D, Saleem M, Mallick B, Jha S (2018). Silver nanoparticles fabricated using medicinal plant extracts show enhanced antimicrobial and selective cytotoxic propensities. IET Nanobiotechnol.

[287] Varalakshmi KN, Sangeetha CG, Samee US, Irum G, Lakshmi H, Prachi SP (2011). In vitro safety assessment of the effect of five medicinal plants on human peripheral lymphocytes. Trop J Pharm Res.

[288] Składanowski M, Golinska P, Rudnicka K, Dahm H, Rai M (2016). Evaluation of cytotoxicity, immune compatibility and antibacterial activity of biogenic silver nanoparticles. Med Microbiol Immunol (Berl).

[289] AshaRani PV, Low Kah Mun G, Hande MP, Valiyaveettil S (2009). Cytotoxicity and genotoxicity of silver nanoparticles in human cells. ACS Nano.

[290] Hussain SM, Hess KL, Gearhart JM, Geiss KT, Schlager JJ (2005). In vitro toxicity of nanoparticles in BRL 3A rat liver cells. Toxicol Vitro Int.

[291] Burd A, Kwok CH, Hung SC, Chan HS, Gu H, Lam WK, Huang L (2007). A comparative study of the cytotoxicity of silver-based dressings in monolayer cell, tissue explant, and animal models. Wound Repair Regen..

[292] Poon VKM, Burd A (2004). In vitro cytotoxity of silver: implication for clinical wound care. Burns J Int Soc Burn Inj.

[293] Walker M, Parsons D (2012). The biological fate of silver ions following the use of silver-containing wound care products—a review. Int Wound J.

[294] Pratsinis A, Hervella P, Leroux J-C, Pratsinis SE, Sotiriou GA (2013). Toxicity of silver nanoparticles in macrophages. Small.

[295] Mlalila NG, Swai HS, Hilonga A, Kadam DM (2017). Antimicrobial dependence of silver nanoparticles on surface plasmon resonance bands against Escherichia coli. Nanotechnol Sci Appl.

[296] Riddick TM (1968). Control of colloid stability through zeta potential.

